# Turning Males On: Activation of Male Courtship Behavior in *Drosophila melanogaster*

**DOI:** 10.1371/journal.pone.0021144

**Published:** 2011-06-22

**Authors:** Yufeng Pan, Carmen C. Robinett, Bruce S. Baker

**Affiliations:** Janelia Farm Research Campus, Howard Hughes Medical Institute, Ashburn, Virginia, United States of America; Freie Universitaet Berlin, Germany

## Abstract

The innate sexual behaviors of *Drosophila melanogaster* males are an attractive system for elucidating how complex behavior patterns are generated. The potential for male sexual behavior in *D. melanogaster* is specified by the *fruitless* (*fru*) and *doublesex* (*dsx*) sex regulatory genes. We used the temperature-sensitive activator dTRPA1 to probe the roles of *fru^M^*- and *dsx*-expressing neurons in male courtship behaviors. Almost all steps of courtship, from courtship song to ejaculation, can be induced at very high levels through activation of either all *fru^M^* or all *dsx* neurons in solitary males. Detailed characterizations reveal different roles for *fru^M^* and *dsx* in male courtship. Surprisingly, the system for mate discrimination still works well when all *dsx* neurons are activated, but is impaired when all *fru^M^* neurons are activated. Most strikingly, we provide evidence for a *fru^M^*-independent courtship pathway that is primarily vision dependent.

## Introduction

A central goal of neuroscience is to understand in molecular detail how neural circuits function to permit individuals to perceive the world and execute specific behaviors based on those perceptions. Innate behaviors in model organisms have attracted substantial interest in this regard as they offer the possibility of applying a variety of neurogenetic tools to address these issues.

A premier biological system that is a center of such efforts is male sexual behavior in the fly *Drosophila melanogaster*. Male sexual behavior in this species, like in many species, is an attractive behavior for study because it is robust, and hence Drosophila male courtship behavior and the neuronal circuitry that subserves it have been the subjects of extensive studies (reviews [1]–[6]). In Drosophila, male courtship is largely innate and consists of a complex series of ordered behaviors that includes orienting, following, tapping, singing (wing extension and vibration), licking, abdomen bending, attempted copulation, copulation, and culminates with ejaculation. The behaviors prior to copulation convey visual, olfactory, gustatory, auditory, and tactile cues that allow males and females to recognize and evaluate potential mating targets and drive the progression of courtship from one behavior to the next. A male will not execute these courtship behaviors (especially the later steps) unless he senses a potential mate. Indeed, throughout the animal kingdom there is a strong linkage between the percept of a potential mate and the display of male sexual behavior. The nature of the coupling between these two processes is largely unknown.

Genetically, *D. melanogaster* male courtship behavior is largely dependent on the sex-specific functions of the genes *fruitless* and *doublesex*, which are the terminal genes of the sex determination hierarchy that together specify nearly all aspects of somatic sexual development including the potential for male sexual behavior [2], [3], [5], [7]–[9]. The pre-mRNAs from both *dsx* and the P1 promoter of *fru* are sex-specifically spliced to yield male- and female-specific DSX proteins (DSX^M^ and DSX^F^, respectively) and male-specific FRU^M^ proteins (in females the homologous *fru* transcripts are not translated [10], [11]). The FRU^M^ proteins, which are expressed post-mitotically, are found exclusively in a dispersed subset of *ca.* 2000 CNS and PNS neurons (*ca.* 2% of the CNS neurons) [10], [12]–[15], whereas *dsx* is expressed in approximately 700 CNS neurons, about 300 in the brain, 60 in the thoracic ganglia, and the majority located in the abdominal ganglion [16]–[19]. There is a partial overlap in the neurons that express *fru^M^* and *dsx*. In particular, the majority of brain and thoracic ganglion neurons expressing *dsx* also express *fru^M^*.

Our current understanding suggests that *fru^M^* has the predominant role in specifying the potential for male sexual behavior. Thus *fru^M^* expression in the appropriate neurons is both necessary and sufficient to confer the potential for nearly all aspects of male sexual behavior [4], [12], [20]. Moreover, the *fru^M^*-expressing neurons are dedicated to these functions as silencing these neurons produces defects only in sexual behaviors [12], [13]. Taken together these findings support the proposal that the *fru^M^*-expressing neurons comprise a circuitry responsible for, and dedicated to, male sexual behaviors [2], [7]. These and other findings further suggest that the *fru^M^*-specified neural circuitry may control all aspects of courtship behavior, from recognizing a target, through the coordination and execution of the steps of courtship itself.

The neurons expressing the *fru-P1* promoter are also important for other sex-specific Drosophila behaviors. These include male- and female-specific patterns of aggression [21]–[23]. Further, in females, at least some of the neurons in which *fru-P1* is expressed are important for female reproductive behaviors [24]–[27].

In addition to regulating most aspects of somatic sexual differentiation outside of the nervous system [28], [29], *dsx* has been shown to play an important role in the generation of sexually dimorphic numbers of neurons in parts of the CNS and PNS [17], [18], [30]–[32]. In terms of a role in male courtship behavior, while *dsx* has been shown to be important for the production of courtship song (*dsx* null mutants lack sine song), other reported behavioral effects of the absence of *dsx* function are a general decrement in the level of male courtship [33].

One approach to probe the functional logic of the *fru^M^* and *dsx* courtship circuitries is to examine the behavioral consequences of activating those circuits using neurogenetic tools. A recent study using optogenetic technology to activate all *fru^M^* neurons showed that courtship song and abdomen bending could be elicited in headless flies [34]. However, this manipulation failed to induce song or any other individual courtship behaviors in intact males at significant levels.

Here, we used a warmth-activated cation channel, Drosophila TRPA1 (dTRPA1 [35], [36]), to activate via a temperature shift either the entire *fru^M^* or entire *dsx* circuitry. Solitary males in which these circuits were activated showed robust levels of nearly all courtship behaviors. We analyzed these behaviors in both intact and headless males, and also in intact males with a variety of courtship targets. Our results indicate that the *fru^M^* circuitry is responsible for both mate recognition and execution of the later steps of courtship. The *dsx* circuitry is mainly involved in execution of the later steps of courtship. We provide evidence for a *fru^M^*-independent courtship pathway that is primarily vision dependent.

## Results

### Activation of *fru^M^* or *dsx* neurons is sufficient to induce male courtship

Although the functions of the *fru* and *dsx* genes in the *D. melanogaster* nervous system have been shown to be critical for establishing the circuitry that houses the potential for male courtship behavior, relatively little is yet known about how that circuitry functions [2], [3], [5], [8], [9]. Here we probe the overall functional status of the male courtship circuitry by acutely activating either all *fru^M^* or all *dsx* neurons in adults and characterizing the courtship behavioral consequences of those manipulations.

**Figure 1:**
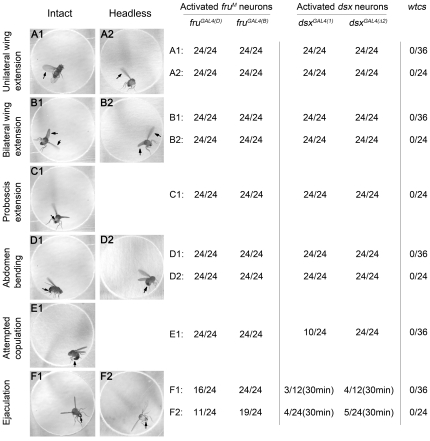
Activation of all *fru^M^* or all *dsx* neurons triggers almost all steps of male courtship rituals. (A–F) Video stills of an intact male (A1–F1) or headless male (A2–F2) expressing *dTrpA1* under *fru^GAL4(B)^* control. Videos were taken at 29°C. All late steps of male courtship behavior can be induced in such condition, including wing extension (A1–2 for unilateral wing extension and B1–2 for bilateral wing extension), proboscis extension (C1; not in headless males), abdomen bending (D1–2), attempted copulation (E1; not found in headless males) and ejaculation (F1–2). The distinction between abdomen bending and attempted copulation is that the latter is a momentary thrusting action during which a male fully curls its abdomen. These behaviors can also be elicited in solitary males by activating all *dsx* neurons. The right panel indicates the fraction of males tested that show the indicated behavior in 15 min after a shift from 22°C to 29°C. Control wild-type males did not display any courtship-like behavior in the observation period.

To simultaneously activate all *fru^M^* neurons we used *fru^GAL4(B)^* [12] and *fru^GAL4(D)^* [13], independent targeted insertions of *GAL4* into the P1 transcription unit of the *fru* gene, to drive expression of the temperature-gated activator *dTrpA1* in *UAS*-*dTrpA1*/+; *fru^GAL4^*/+ males. Solitary males of these genotypes did not display any courtship-like behavior at the permissive temperature (22°C) (Table S1). However, transfer of these solitary males to a temperature that activates *dTrpA1* (29°C) rapidly elicited almost all steps of male courtship behavior in every male (Figure 1, Movie S1). Courtship steps observed included unilateral wing extension and vibration, proboscis extension, abdomen bending, attempted copulation (for the distinction between abdomen bending and attempted copulation, see Materials and Methods, and also Movie S2), and ejaculation (Figure 1). One elicited behavior that is not normally characteristic of courtship was bilateral wing extension and vibration (for evidence that this behavior is also courtship related see below). There were no obvious qualitative differences in the patterns of courtship behaviors displayed by the two experimental *fru^GAL4^* genotypes (Figure 1). Recently, it was independently reported that the expression of *dTrpA1* driven by either *fru^GAL4(D)^* [37] or *fru^NP21^* [38] elicits some male courtship behaviors.

To explore the role of *dsx*-expressing neurons in male courtship behavior, we used two targeted insertions of *GAL4* into the *dsx* gene, *dsx^GAL4(1)^* [19] and *dsx^GAL4(^* ^Δ^ *^2)^* (see Materials and Methods), which we refer to collectively as *dsx^GAL4^*, to examine the consequences of activation of the *dsx-*expressing neural circuitry in *UAS*-*dTrpA1*/+; *dsx^GAL4^*/+ solitary males. These males did not display any obvious courtship-like behavior at the permissive temperature for *dTrpA1* (22°C) (Table S1). Strikingly, shifting these males to 29°C elicited an array of courtship phenotypes qualitatively similar to those elicited upon activation of *fru^M^* neurons (Figure 1 and Movie S3). Thus, these solitary *UAS*-*dTrpA1*/+; *dsx^GAL4^*/+ males showed unilateral wing extension and vibration, proboscis extension, abdomen bending, and attempted copulation, as well as bilateral wing extension (Figure 1). Unlike *fru^GAL4^* males, they did not ejaculate in the 15 min observation period, but a few ejaculated in a longer observation period (30 min). The two *dsx^GAL4^* drivers elicited qualitatively similar patterns of courtship behaviors (Figure 1).

As the courtship behaviors described above were manifested by solitary males, it may be asked whether these behaviors are really courtship. That they are courtship behaviors is strongly supported by the finding that during wing vibration these males produced courtship song (see below). In addition, use of a sperm-specific *GFP* (*don juan-GFP* [39]) revealed that the “ejaculates” of these solitary males contain labeled sperm (Figure S1).

As controls for the above experiments, we tested wild-type males (Figure 1) as well as males with *fru^GAL4(B)^*, *fru^GAL4(D)^*, *dsx^GAL4(1)^*, *dsx^GAL4(^* ^Δ^ *^2)^*, or *UAS*-*dTrpA1* alone via temperature shifts identical to those described above (Table S2). None of these control genotypes showed any courtship-like behavior at 29°C. We also tested males at 25°C and 27°C and found that the behavioral consequences of activating all *fru^M^* or all *dsx* neurons were sensitive to temperature, as expected (Table S1).

It has been shown that male-like courtship behavior can be elicited in females by optical activation of P2X_2_ in the neurons homologous to those that express *fru^M^* in males [34]. Similarly, *dTrpA1*-mediated activation of all *fru^M^*-expressing neurons in females (driven by either *fru^GAL4^*) at 29°C was sufficient to induce unilateral wing extension (Figure S2A); however, these females produced atypical songs (Figure S2B–C) compared to male songs (Figure S3), as was also observed by Clyne and Miesenbock [34]. Activating all *dsx*-expressing neurons in females via *dTrpA1* did not elicit obvious male-like courtship behavior within 15 min at 29°C.

### Induction of courtship behavior in headless males

A male identity of some neurons in the ventral nerve cord (VNC) is necessary for the execution of certain aspects of male courtship behavior [30], [40]–[42], and is even sufficient for song if appropriately stimulated [34]. We therefore asked whether courtship behaviors could be elicited by a shift of *UAS-dTrpA1/+*; *fru^GAL4^/+* and *UAS-dTrpA1/+*; *dsx^GAL4^/+* headless males to the restrictive temperature of 29°C. Activation of *fru^M^* or *dsx* neurons in headless males produced an array of courtship phenotypes almost identical to those observed in intact males of these genotypes: unilateral wing extension and vibration, abdomen bending, and ejaculation (ejaculation was scored for 30 min on activation of *dsx* neurons, and all other phenotypes for 15 min; Figure 1). As seen with intact males of these genotypes, these headless males also displayed bilateral wing extension and vibration, which is not typically associated with courtship (but see below). Unlike what was observed in intact males, these headless males never showed attempted copulation, suggesting that attempted copulation may be under direct descending control of neurons in the brain. These results provide direct evidence that certain *fru^M^*-expressing neurons as well as certain *dsx*-expressing neurons in the VNC, in their active state, are sufficient to promote execution of wing extension and vibration, abdomen bending, and ejaculation. A caveat to these conclusions is that severed axons descending from the brain to the VNC may have been sufficiently intact that they could be activated by *dTrpA1* at the restrictive temperature; nevertheless, wing extension and abdomen bending could still be elicited by *dTrpA1* activation in males even 4 days after decapitation (for details, see Materials and Methods).

### Kinetics of wing extension and abdomen bending in intact and headless males

**Figure 2 pone-0021144-g002:**
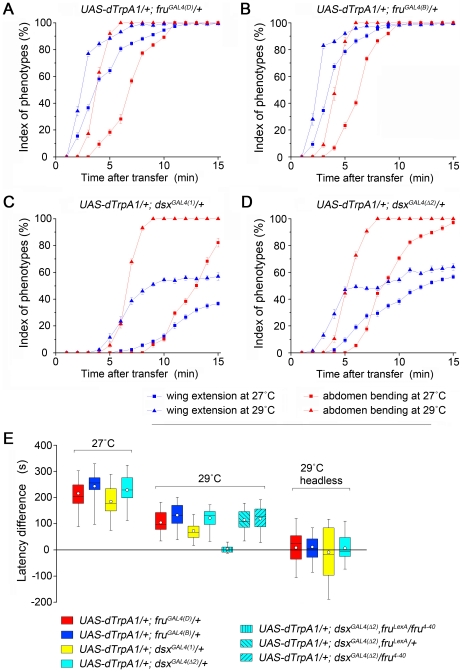
The sequence of courtship events is maintained in *dTrpA1*-mediated courtship behaviors and dependent on FRU^M^. Two prominent steps of courtship behavior, wing extension and abdomen bending, were analyzed in each minute after transfer from permissive temperature (22°C) to restrictive temperatures (27°C and 29°C) for 15 min. (A–D) Indices of indicated phenotype (blue: wing extension; red: abdomen bending; rectangle: 27°C; triangle: 29°C) are shown for each minute on continuous activation driven by *fru^GAL4(D)^* (A), *fru^GAL4(B)^* (B), *dsx^GAL4(1)^* (C) and *dsx^GAL4(^* ^Δ^ *^2)^* (D). For each one-minute sample these indices were calculated as percentage of the period during which males showed an indicated phenotype. For each genotype, the index increased over time. (E) Activation of all *fru^M^* or *dsx* neurons induced ordered courtship behaviors. Box plots indicate how long the first abdomen bending occurred after the first wing extension. Circles indicate mean values. The middle line represents the median and the ends of the vertical lines indicate the minimum and maximum. The upper and lower edges of the boxes represent the first and third quartiles. The data indicates that wing extension is always induced before abdomen bending in solitary males regardless of temperatures (27°C and 29°C) or drivers (two *fru^GAL4^* drivers and two *dsx^GAL4^* drivers). Such order is dependent on FRU^M^ as activating all *dsx* neurons in *fru^M^* null males induces wing extension and abdomen bending simultaneously. The order also lost in headless males for all four drivers. *n* = 12∼24 for each. Error bars in A–D indicate SEM.

While the above experiments established that many aspects of male courtship behavior could be elicited by the *dTrpA1*-mediated activation of either *fru^M^-* or *dsx-*expressing neurons in both intact and headless males, they did not speak to the intensities with which these males courted. To assess how robust courtship was in these males, we examined how two prominent courtship components, wing extension and abdomen bending, were manifested immediately after shifting these males to restrictive temperatures (both 27°C and 29°C for intact males, Figure 2A–D; and 29°C for headless males, Figure S4A–D). Wing extension and abdomen bending were both manifested in intact males within 2–5 min at 29°C with both the *fru^GAL4^* and *dsx^GAL4^* drivers (Figure 2A–D). By 10 min at 29°C nearly all males of these four genotypes were almost continuously displaying abdomen bending. In contrast, while wing extension was also displayed almost continuously in nearly all *fru^GAL4^* males by 10 min at 29°C, wing extension for males expressing either *dsx^GAL4^* driver at 29°C plateaued with an index of 50–60%. Manifestation of both wing extension and abdomen bending was generally slower at lower temperature of 27°C. With headless males, the kinetics of wing extension and abdomen bending at 29°C were similar to but more variable (larger SEM values) than those of intact males (Figure S4A–D), suggesting a role of the brain in regulating these behaviors. Additional differences in courtship behavior between intact and headless males were seen in more detailed analyses of courtship sequence, wing usage and courtship song (see below).

### *fru^M^* function in the brain promotes proper ordering of courtship behaviors

The wild-type male Drosophila courtship ritual, especially when first initiated, is largely a dependent action pattern with the steps of courtship occurring in a fixed sequence [43]. This dependent action pattern requires *fru^M^* function in a group of *fru^M^*-expressing neurons in the suboesophageal ganglion that project through the median bundle, as disruption of *fru^M^* function in these neurons results in the later steps of courtship (wing extension and vibration, licking, and attempted copulation) occurring simultaneously [44]. We were therefore particularly interested in what effect, if any, the activation of all *fru^M^* or all *dsx* neurons would have on the sequence with which the steps of courtship were executed.

To address this question, we examined intact solitary *UAS-dTrpA1/+*; *fru^GAL4^/+* and *UAS-dTrpA1/+*; *dsx^GAL4^/+* males for the temporal sequence of appearance of wing extension, abdomen bending and attempted copulation upon a shift from permissive temperature (22°C) to a restrictive temperature (either 27°C or 29°C) (Figure 2E). For all males tested, attempted copulation, when observed, was always elicited after wing extension and abdomen bending (data not shown, but see Movie S1 and S3). Furthermore, wing extension and abdomen bending did not appear simultaneously upon the activation of all *fru^M^* neurons, but instead appeared in their normal temporal sequence wherein wing extension always appeared before abdomen bending at a population level, as well as the level of individual males, at both 27°C and 29°C (Figure 2E and Movie S1). Activation of all *dsx* neurons also induced wing extension and abdomen bending in their normal temporal sequence at both 27°C and 29°C (Figure 2E and Movie S3).

Factors that should contribute to the latencies of wing extension and abdomen bending (mostly 2–5 min) are: (1) the time it takes to warm from the permissive to the restrictive temperature; (2) differences in the thresholds for functionally significant activation of the relevant neurons that control wing extension and abdomen bending; (3) the time from functionally significant activation of *fru^M^* or *dsx* neurons to execution of behavior. The first factor should be the same with respect to each step, and the third factor is likely trivially short relative to the observational time frames. Thus the latency difference of wing extension and abdomen bending may be the result of different thresholds for functionally significant activation in the two neuronal populations. To further examine this topic, we placed males directly on a 29°C plate in order to shift them to the restrictive temperature more rapidly (for details, see Materials and Methods). In this context, wing extension and abdomen bending were initiated much more quickly but still in their normal sequence (latencies of wing extension: 6.8±0.7 s, 6.7±0.9 s, 24.5±1.9 s, 19.2±1.4 s using *fru^GAL4(D)^*, *fru^GAL4(B)^*, *dsx^GAL4(1)^* and *dsx^GAL4(^* ^Δ^ *^2)^* drivers, respectively; latencies of abdomen bending: 16.3±1.7 s, 13.3±1.9 s, 44.6±3.7 s, 30.3±2.7 s, respectively). Thus a longer delay for initiation of abdomen bending than for initiation of wing extension was seen independent of the speed with which the temperature was ramped.

It has been speculated that the subset of *fru^M^-*expressing median bundle neurons implicated in sequencing the steps of courtship might bring this about by setting progressively higher thresholds of male arousal/excitation for successive steps of courtship [44]. If this mechanism were operative here, one would expect that the latency difference (courtship order) would be lost in headless males, which lack median bundle neurons. Indeed, we found that latencies for wing extension and for abdomen bending (following a shift from 22°C to 29°C) were not significantly different in headless males for each *GAL4* used (Figure 2E). Consistent with this finding, we also found that activating all *dsx* neurons in intact *fru^M^* null males (*fru^LexA^/fru^4–40^*; with *fru^LexA^/+* and *fru^4–40^/+* as controls) induced wing extension and abdomen bending simultaneously (Figure 2E and Movie S4). These results indicate that FRU^M^ enables certain neurons in the brain to carry out their function in the ordering of courtship steps, and activating *dTrpA1* in these neurons does not affect that function.

### On the control of unilateral vs. bilateral wing usage

**Figure 3:**
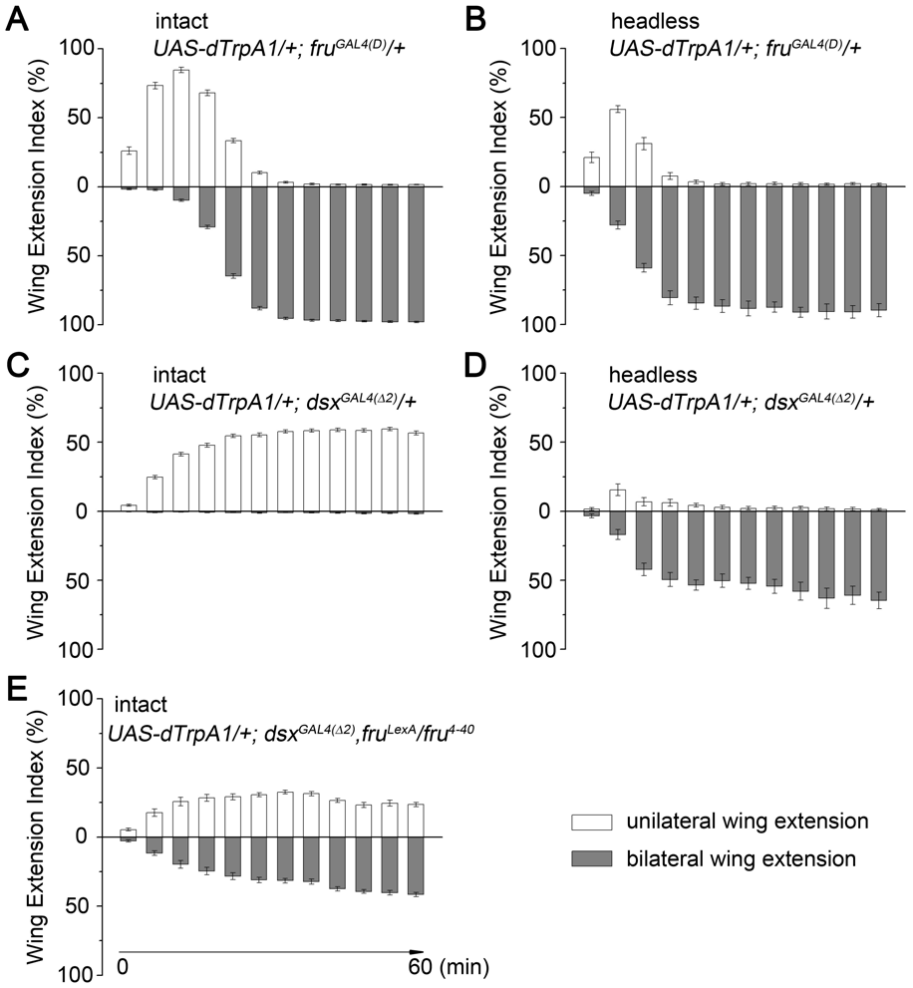
Wing extension patterns in intact and headless males. Individual males were transferred from 22°C to 27°C and recorded for 1 hour. For each male, indices of wing extension (for definition, see Materials and Methods), for either unilateral wing extension (white) or bilateral wing extension (gray), were analyzed for every 5-min period (i.e., 1–5 min, 6–10 min, and the last, 56–60 min; thus there are in total 12 indices for unilateral wing extension and another 12 indices for bilateral wing extension). Each bar represents a 5-min period. (A–B) Wing extension indices in intact (A) and headless (B) *UAS-dTrpA1/+*; *fru^GAL4(D)^/+* males at 27°C. The transition from unilateral to bilateral wing extension occurs more quickly in headless males. (C–D) Wing extension indices in intact (C) and headless (D) *UAS-dTrpA1/+*; *dsx^GAL4(^* ^Δ^ *^2)^/+* males at 27°C. Activation of all *dsx* neurons produces consistent unilateral wing extension in intact males, but dominant bilateral wing extension in headless males. (E) Wing extension indices in intact *UAS-dTrpA1/+*; *dsx^GAL4(^* ^Δ^ *^2)^*, *fru^LexA^/fru^4–40^* males at 27°C. Loss of *fru^M^* function partially disrupts the wing extension pattern in intact males resulting in approximately equal unilateral and bilateral wing extensions. *n* = 8∼12 for each. Error bars indicate SEM.

One unexpected phenotype we found upon activation of all *fru^M^* or all *dsx* neurons was the induction of bilateral wing extensions in addition to the unilateral wing extensions that are normal for courtship. These two patterns of wing usage varied across time and by genotype. We determined wing usage patterns in a 1-hour observation period after a shift to 27°C (Figure 3A–E; each bar represents wing extension index in a 5-min period). During the first 20 min following activation of *fru^M^* neurons, most wing extension was unilateral (*fru^GAL4(D)^*, Figure 3A; and *fru^GAL4(B)^*, Figure S5A). However, continued activation resulted in bilateral wing extension becoming the dominant and ultimately the exclusive mode of wing usage. The transition from unilateral to bilateral wing extension occurred somewhat more quickly in headless males (*fru^GAL4(D)^*, Figure 3B; and *fru^GAL4(B)^*, Figure S5B).

In contrast to what was seen with *fru^GAL4^*, activating *dsx* neurons induced consistent unilateral wing extension in intact males throughout the 1-hour observation period (*dsx^GAL4(1)^*, Figure S5C; and *dsx^GAL4(^* ^Δ^ *^2)^*, Figure 3C). Strikingly, activating *dsx* neurons in headless males (*UAS*-*dTrpA1/+*; *dsx^GAL4^/+*) induced a pattern of exclusively bilateral wing usage (*dsx^GAL4(1)^*, Figure S5D; and *dsx^GAL4(^* ^Δ^ *^2)^*, Figure 3D). These results show that neurons in the brain are involved in determining wing extension patterns. The exclusively unilateral pattern of wing usage seen when *dsx* neurons were activated in intact males (*UAS*-*dTrpA1/+*; *dsx^GAL4(^* ^Δ^ *^2)^/+*) was dependent on *fru^M^* function, since when *dsx* neurons were activated in *fru^M^* null males (*UAS*-*dTrpA1/+*; *dsx^GAL4(^* ^Δ^ *^2)^*, *fru^LexA^/fru^4–40^*) the pattern switched to approximately equal unilateral and bilateral wing usage (Figure 3E). Since the absence of *fru^M^* function resulted in both unilateral and bilateral wing extension, we conclude that the net function of wild-type *fru^M^* is to suppress bilateral wing usage.

### Sociality-dependent courtship song properties

As males in which *fru^M^-* or *dsx-*expressing neurons had been activated extended and vibrated their wings, we examined whether these wing vibrations were associated with the production of courtship song and, if so, the properties of their songs, and the roles of *fru^M^* and *dsx* in song production. Courtship song in *D. melanogaster* is composed of pulse and sine components with pulse song being characterized by the interpulse interval (IPI) and sine song by its frequency [45], [46].

**Figure 4:**
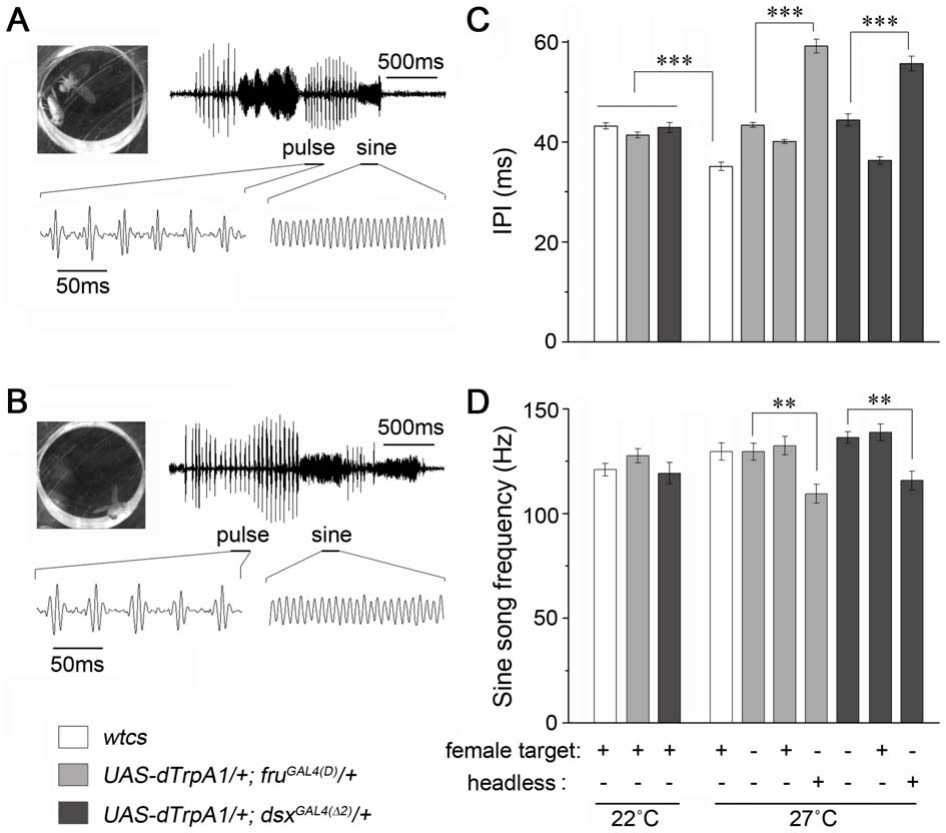
Courtship song properties of intact and headless males. (A–B) Courtship song samples from a pair of wild-type *Canton S* (*wtcs*) flies at 27°C (A), and from a solitary male with all *fru^M^* neurons being activated (*UAS*-*dTrpA1*/+; *fru^GAL4(D)^*/+) (B). Both samples contain sine and pulse components. (C–D) Interpulse interval (IPI) of the pulse song (C) and frequency of the sine song (D). All male-female pairs tested at 22°C showed no difference from each other, and their IPIs are higher than wild-type pairs at 27°C (*p*<0.001, ANOVA). IPIs acquired in solitary males at 27°C are also significantly higher than wild-type pairs (white) at 27°C; further more, IPIs in headless males are higher than those in intact males (*p*<0.001 for both *UAS*-*dTrpA1*/+; *fru^GAL4(D)^*/+ (light gray) and *UAS*-*dTrpA1*/+; *dsx^GAL4(^* ^Δ^ *^2)^*/+ (gray) males, two sample t-test). The introduction of a female target at 27°C partially (to *UAS*-*dTrpA1*/+; *fru^GAL4(D)^*/+ male) or fully (to *UAS*-*dTrpA1*/+; *dsx^GAL4(^* ^Δ^ *^2)^*/+ male) restored IPI to wild type level. Sine song frequencies were lower in headless males (*p*<0.01, two sample t-test). *n* = 7–8 for each. **p<0.01 and ***p<0.001. Error bars indicate SEM.

We recorded courtship songs produced by males in three contexts: (1) control wild-type male-female pairs (Figure 4A and S3A); (2) solitary males with either the *fru^M^* (Figure 4B and S3F–J) or *dsx* circuitry activated (Figure S4B–E); and (3) headless males with either *fru^M^* or *dsx* circuitry activated. As additional controls, we tested *UAS-dTrpA1/+*; *fru^GAL4^/+* and *UAS-dTrpA1/+*; *dsx^GAL4^/+* males paired with virgin females at the permissive temperature (22°C). Wild type, *UAS*-*dTrpA1*/+; *fru^GAL4(D)^*/+, and *UAS*-*dTrpA1*/+; *dsx^GAL4(^* ^Δ^ *^2)^*/+ males when paired with female targets at 22°C produced song with comparable properties (Figure 4C–D). As the IPI in wild-type males was significantly lower at higher temperature (IPIs are 43.1±0.6 ms and 35.2±0.8 ms at 22°C and 27°C, respectively, *p*<0.001), in the experiments described below we compared IPIs from different genotypes at the same temperature.

Activating the *fru^M^* circuitry by shifts of solitary *UAS*-*dTrpA1*/+; *fru^GAL4(D)^*/+ males to 27°C initially elicited alternating sine and pulse songs (Figure 4B and S3H). However, after a few minutes these males began producing sine and pulse songs simultaneously (Figure S3I–J). Similar results were obtained by using *fru^GAL4(B)^* (Figure S3F–G). Two possibilities as to the origin of simultaneous sine and pulse song suggest themselves: (1) sine and pulse songs are generated by distinct, mutually inhibitory neuronal substrates, but continuous activation of both circuits breaks down the inhibition and leads to the production of sine and pulse songs at the same time; and (2) coincident generation of sine song by one wing and pulse song by the other. We thus recorded songs from *UAS*-*dTrpA1*/+; *fru^GAL4(D)^*/+ males with only one wing (either left or right), and were still able to detect simultaneous sine and pulse songs (data not shown), suggesting that these two song components were produced through separate circuits.

Surprisingly, activating the *dsx* circuitry in solitary males via the two *dsx^GAL4^* drivers produced discordant results. Activating the circuitry by using *dsx^GAL4(^* ^Δ^ *^2)^* triggered both sine and pulse components (Figure S3E) similar to what is seen in wild type, while activating the *dsx* circuitry via the *dsx^GAL4(1)^* driver initiated predominantly sine song, with sporadic pulses (Figure S3B). When we tested *UAS-dTrpA1*/+; *dsx^GAL4(1)^*/+ males with female targets at 22°C and 27°C, they showed both sine and pulse components (Figure S3C–D). The finding that solitary *UAS-dTrpA1*/+; *dsx^GAL4(1)^*/+ males produced almost exclusively sine song at 27°C is intriguing in light of the previous observation that *dsx* loss-of-function mutants are specifically defective in the production of sine song [33]. These results further suggest that sine and pulse songs are programmed by distinct neural circuits: *dsx^GAL4(1)^* may only target the circuit for sine song. Whether this distinction results from differences in the expression patterns of the two drivers, which differ modestly in some anatomical regions (Table S3), is yet to be determined. However, in males with activated *dsx^GAL4(1)^* neurons, the presence of female cues enables the production of pulse song through activation of additional neuronal components.

We further examined the properties of courtship song produced by solitary *UAS*-*dTrpA1*/+; *fru^GAL4(D)^*/+ and *UAS*-*dTrpA1*/+; *dsx^GAL4(^* ^Δ^ *^2)^*/+ males during the first 3 min after the initiation of song at 27°C (Figure 4C–D). Activating all *fru^M^* or *dsx* neurons in intact solitary males produced pulse song with IPIs (43.5±0.5 ms and 44.4±1.2 ms for *fru^GAL4(D)^* and *dsx^GAL4(^* ^Δ^ *^2)^*, respectively) that were longer than those of wild-type males at 27°C (35.2±0.8 ms; *p*<0.001 for both *fru^GAL4(D)^* and *dsx^GAL4(^* ^Δ^ *^2)^*). Such activation in headless solitary males induced pulse song with IPIs that were even longer than those of intact males of the same genotype (59.2±1.4 ms and 55.7±1.5 ms for *fru^GAL4(D)^* and *dsx^GAL4(^* ^Δ^ *^2)^*, respectively, *p*<0.001 for both drivers, Figure 4C). Headless males also showed lower sine song frequencies than did intact males (*p*<0.01 for both *fru^GAL4(D)^* and *dsx^GAL4(^* ^Δ^ *^2)^* drivers, Figure 4D). When intact *UAS*-*dTrpA1*/+; *fru^GAL4(D)^/+* males were paired with female targets at 27°C, the IPI (40.1±0.4 ms) was reduced toward the level seen in wild-type male-female pairs. The presence of a female with an intact *UAS*-*dTrpA1*/+; *dsx^GAL4(^* ^Δ^ *^2)^*/+ male fully restored the IPI to the wild-type level at 27°C (36.3±0.7 ms vs 35.2±0.8 ms for *UAS*-*dTrpA1*/+; *dsx^GAL4(^* ^Δ^ *^2)^*/+ and wild-type males; *p*>0.05). Thus, males with either their *fru^M^* or *dsx* circuitry activated are able to sense females and alter their behavior in response to this sensory input.

### Discrimination between potential courtship targets by males with *fru^M^* neurons activated

The *fru^M^-* and *dsx*-specified neuronal circuitry is important not only for the manifestation of all male courtship behaviors, but also for the perception of sensory cues that allow recognition of potential mates and thereby elicit courtship behaviors. Thus, visual and olfactory cues are important for the initial recognition of and discrimination between potential mates, while additional gustatory, auditory and tactile cues contribute to later steps of courtship.

To determine whether males with activated *fru^M^* or *dsx* circuitry could recognize and discriminate between potential courtship targets, we examined the courtship behavior of test males paired with male or female *D. melanogaster*, as well as females of two other Drosophila species: *D. yakuba* and *D. mojavensis*. Given that activation of the *fru^M^* circuitry or the *dsx* circuitry led to high levels of courtship behaviors in solitary males (in the absence of a courtship target), we quantitated the amount of courtship that was directed at the target as a percentage of overall courtship behavior and defined two different courtship indices (CIs): courtship to a target (CI_target_, the fraction of the observation period when the male is courting a target), and total courtship output (CI_output_, used when *fru^M^* or *dsx* neurons are activated via *dTrpA1*, and is the fraction of the observation period when the male is displaying courting behaviors, independent of whether they are directed at the target).

**Figure 5:**
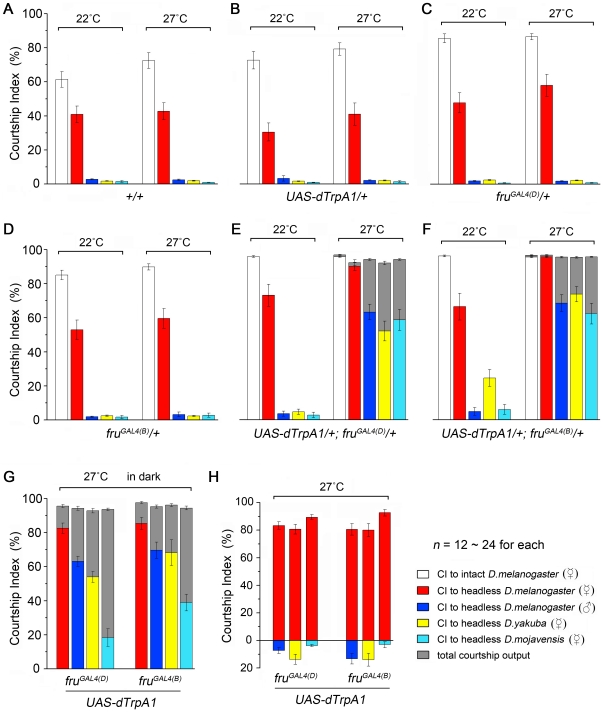
*fru^M^* neurons in courtship promotion and recognition. (A–F) Courtship indices of males of indicated genotypes paired with *D. melanogaster* females (white), headless *D. melanogaster* females (red), headless *D. melanogaster* males (blue), headless females of *D. yakuba* (yellow) or *D. mojavensis* (cyan). (A–D) Wild-type males (A) and males with *UAS-dTrpA1* (B) or *fru^GAL4^* alone (C–D, *fru^GAL4(D)^* and *fru^GAL4(B)^* respectively) directed strong courtship to intact and headless *D. melanogaster* females, but little to inappropriate targets at either 22°C or 27°C. (E–F) Activation of all *fru^M^* neurons induced consistently high level of courtship outputs (gray), with the highest levels being directed at *D. melanogaster* females, and less elevated levels directed at headless *D. melanogaster* males and headless females of *D. yakuba* and *D. mojavensis*. (G) Courtship indices of *fru^M^*-activated males paired with headless *D. melanogaster* females and males, and headless females of *D. yakuba* and *D. mojavensis* in the dark. (H) Courtship indices of *fru^M^*-activated males paired with two targets in a competitive assay. One target is always a headless *D. melanogaster* female, and the other is a headless *D. melanogaster* male or headless female of *D. yakuba* or *D. mojavensis*. For both *fru^GAL4^* drivers, these males prefer to court conspecific females. *n* indicates number of males tested. Error bars indicate SEM.

Control wild-type males strongly courted intact *D. melanogaster* females at both 22°C and 27°C, and courted headless *D. melanogaster* females at somewhat reduced levels (Figure 5A). These males rarely court headless males, headless *D. yakuba* females, or headless *D. mojavensis* females (referred to “inappropriate targets” in the following). Control males with *UAS-dTrpA1* alone, or either *fru^GAL4(D)^* or *fru^GAL4(B)^* alone had courtship patterns similar to wild type (Figure 5B–D), as did *UAS-dTrpA1*/+; *fru^GAL4(D)^*/+ and *UAS-dTrpA1*/+; *fru^GAL4(B)^*/+ males at the permissive temperature (Figure 5E–F).

When *UAS-dTrpA1*/+; *fru^GAL4(D)^/+* and *UAS-dTrpA1*/+; *fru^GAL4(B)^*/+ males were paired with potential courtship targets and all *fru^M^* neurons in these males were activated by a shift to 27°C (prewarmed for 5–10 min to allow activation of *fru^M^* neurons before the introduction of courtship targets), they displayed high levels of total courtship output as reported above (all CI_output_s>90%, Figure 5E–F). However, different potential courtship targets elicited significantly different levels of directed courtship. These males courted *D. melanogaster* females (both intact and headless) intensely (CI_target_s>90%, Figure 5E–F) and copulated with intact females at frequencies comparable to wild-type controls (data not shown, but see Movie S5). These males also courted headless *D. melanogaster* males and headless females of the other species more intensely than did control males (CI_target_s 53–63% for *fru^GAL4(D)^* and CI_target_s 62–73% for *fru^GAL4(B)^*, Figure 5E–F), although the CI_target_s for each *fru^GAL4^* were significantly lower than what they exhibited when paired with intact or headless *D. melanogaster* females (*p*<0.001 for all). This target-directed courtship was not strongly dependent on visual stimuli, since both *UAS-dTrpA1*/+; *fru^GAL4(D)^/+* and *UAS-dTrpA1*/+; *fru^GAL4(B)^*/+ males with *fru^M^* neurons activated also courted both *D. melanogaster* females as well as inappropriate targets in the dark (Figure 5G). A competitive courtship assay using two different targets further demonstrated the males' preference for conspecific females (Figure 5H).

### Discrimination between potential courtship partners by males with *dsx* neurons activated

**Figure 6:**
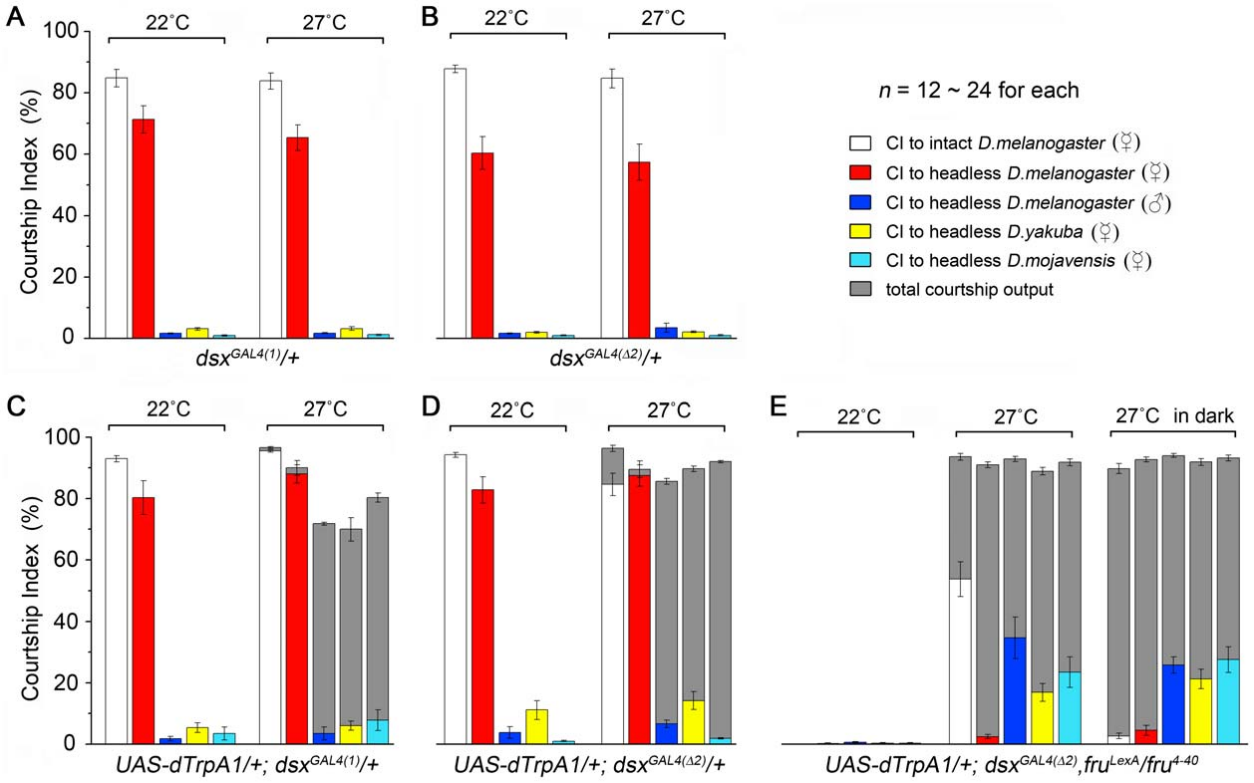
Activation of *dsx* neurons promotes courtship in the absence of FRU^M^. (A–E) Courtship indices of males of the indicated genotypes paired with *D. melanogaster* females (white), headless *D. melanogaster* females (red), headless *D. melanogaster* males (blue), headless females of *D. yakuba* (yellow) and *D. mojavensis* (cyan). (A–B) Males with only *dsx^GAL4^* (either *dsx^GAL4(1)^* or *dsx^GAL4(^* ^Δ^ *^2)^*) but no *UAS-dTrpA1* court *D. melanogaster* females normally, and rarely court other inappropriate targets at either 22°C or 27°C. (C–D) Activation of all *dsx* neurons induced consistently high level of courtship outputs in males at 27°C (gray), independent of the presence of any target; however, these males only directed courtship persistently to *D. melanogaster* female targets. Courtship indices to headless *D. melanogaster* males, headless females of *D. yakuba* and *D. mojavensis* are not significantly different from those acquired at 22°C (*p*>0.05 for all, two sample t-test). (E) Activation of all *dsx* neurons in *fru^M^* null males (*fru^LexA^*/*fru^4–40^*) promoted courtship to intact *D. melanogaster* females in the light but not the dark. These males did not court headless *D. melanogaster* females in light or dark, but they directed courtship to headless males and other species in both the light and the dark. *n* indicates number of males tested. Error bars indicate SEM.

We next assessed the effect of activating *dsx* neurons in males on target-directed courtship. Control males with a copy of either *dsx^GAL4(1)^* or *dsx^GAL4(^* ^Δ^ *^2)^* but no *UAS-dTrpA1* strongly courted *D. melanogaster* females (both intact and headless), but not headless *D. melanogaster* males or headless females of the other species at both 22°C and 27°C (Figure 6A–B). Activating all *dsx* neurons in males at 27°C led to high levels of overall courtship (CI_output_s 70–95%; Figure 6C–D). However, while these males vigorously courted both intact and headless *D. melanosaster* females (CI_target_s 85–95%; Figure 6C–D), they displayed levels of courtship to the inappropriate targets that did not differ significantly from the levels seen at the permissive temperature (Figure 6C–D). The above data indicate that activating *dsx* neurons affects neither a male's ability to recognize conspecific females and direct courtship to them, nor to discriminate between sexes and species.

The fact that males with activated *dsx* neurons showed high courtship outputs but only courted appropriate targets suggested that the system for discriminating between potential courtship targets was still operative in these males. To test whether this system depended on *fru^M^* function, we activated *dsx* neurons in *fru^M^* null males (*fru^LexA^/fru^4–40^*). Such males (*UAS-dTrpA1*/+; *dsx^GAL4(^* ^Δ^ *^2)^*, *fru^LexA^*/*fru^4–40^*) hardly courted at the permissive temperature, which is the expected phenotype of *fru^LexA^/fru^4–40^* males (Figure 6E). Surprisingly, when transferred to the restrictive temperature (27°C) in the light, these males displayed high levels of courtship (all CI_output_s>80%). However, the frequencies with which they directed courtship toward various targets differed from what was seen for *UAS-dTrpA1*/+; *dsx^GAL4(^* ^Δ^ *^2)^*/+ males that were *fru^M+^* (Figure 6E vs. 6C–D). First, courtship of appropriate targets by *UAS-dTrpA1*/+; *dsx^GAL4(^* ^Δ^ *^2)^*, *fru^LexA^*/*fru^4–40^* males was impaired: courtship of intact *D. melanogaster* females was reduced by almost half, and headless *D. melanogaster* females were barely courted at all (CI_target_ = 2.4±0.7%, see Movie S6). That headless *D. melanogaster* females were not courted by males with activated *dsx* circuitry in the absence of *fru^M^* function may reflect a dependence of courtship by these males on motion cues, since headless females move very little.

Supporting the notion that courtship by these males is vision dependent, we found that intact conspecific females were courted by these males only in the light (CI_target_s are 52.9±5.6% and 2.6±0.9% in daylight and in dark, respectively). This suggests that courtship of *D. melanogaster* females by males with activated *dsx* circuitry, but lacking *fru^M^* function, depends almost entirely on visual cues. Second, in the *fru^M^* null background, courtship of inappropriate targets by males with activated *dsx* circuitry was somewhat elevated (Figure 6E; CI_target_s to headless *D. melanogaster* males, headless *D. yakuba* females, and headless *D. mojavensis* females were 34.1±6.7%, 16.6±2.8% and 23.1±4.9%, respectively) compared to what was seen in *UAS-dTrpA1*/+; *dsx^GAL4(^* ^Δ^ *^2)^*/+ males that were *fru^+^* (Figure 6D; CI_target_s to headless *D. melanogaster* males, headless *D. yakuba* females, and headless *D. mojavensis* females were 6.6±1.2%, 14.2±2.9% and 1.9±0.2%, respectively). These findings suggest that *fru^M^*-expressing neurons are responsible, at least in part, for the ability of males with *dsx* circuitry activated to distinguish appropriate from inappropriate courtship objects.

The most striking aspect of these findings is that males completely lacking *fru^M^* function could be induced to court females almost as robustly as wild-type males. The implications of this finding both with respect to *fru^M^*'s function and how the circuitry for male courtship is specified are discussed below.

## Discussion

Recent findings have provided strong support for the proposition that the neuronal populations expressing the *fru* and/or *dsx* genes comprise (a large part of) the circuitry that governs male courtship behavior, as well as other social behaviors in Drosophila (reviews [2], [9]). We found that solitary males in which *dTrpA1* is activated in either all *fru^M^*-expressing or all *dsx*-expressing neurons robustly display most male courtship behaviors—courtship song, licking, abdomen bending, and attempted copulation—within a short time after a shift to the restrictive temperature. Solitary males with *fru^M^* circuitry activated also ejaculated, whereas solitary males with the *dsx* circuitry activated ejaculated less frequently.

Induction of courtship behaviors by *fru^M^*–driven *dTrpA1* is presumably more efficient than the light-activated ion channel effector that was used to stimulate *fru^M^*-expressing neurons as the latter did not elicit male courtship behaviors at significant levels in intact males, although such stimulation of *fru^M^* neurons in headless males did elicit courtship song and a low frequency of abdomen bending [34]. These differences presumably reflect the relative efficiencies of activation in this system by these two approaches.

Detailed analysis of the individual steps of courtship elicited by the activation of *fru^M^* and *dsx* neurons in our experiments has provided insights into the functional properties of the neuronal circuitry governing many aspects of courtship behavior. These comparisons included males that: (1) had *fru^M^* neurons vs. *dsx* neurons activated; (2) were intact vs. headless with either *fru^M^* neurons or *dsx* neurons activated; and (3) had activated *dsx* neurons in a genetic background that had wild-type *fru^M^* function vs. lacked *fru^M^* function.

### General properties of courtship circuitry

Our findings have several implications with respect to the general functional properties of the courtship circuitry. Because we observed courtship behaviors in response to neuronal activation of the *fru^M^* or *dsx* circuitries, we infer that the most proximate *fru^M^*- (or *dsx*-) expressing neuron (or groups of neurons) in the circuitry that leads to execution of a specific aspect of courtship behavior likely has a net activation function with respect to that behavior. As an extension, we infer that any inhibitory neurons that would function to prevent that behavior (in the absence of appropriate stimuli) would likely act further upstream in the courtship circuitry.

Our results also circumscribe the neuronal populations that are likely the sites of the proximate signals that elicit specific courtship behaviors. First, that activation of either all *fru^M^* neurons or all *dsx* neurons elicited the same courtship behaviors suggests two possible explanations for the commonality of their phenotypes. Since *fru^M^* and *dsx* are expressed in partly overlapping sets of neurons in the CNS, one possibility is that these common phenotypes may arise from the subset of neurons that express both *fru^M^* and *dsx*. Alternatively, the *fru^M^* neurons and *dsx* neurons may function in independent, redundant pathways for eliciting courtship behaviors. Distinguishing between these alternatives awaits the developmental of the appropriate neurogenetic tools. Second, our findings that headless males in which either all *fru^M^*-expressing neurons or all *dsx*-expressing neurons have been activated robustly display wing extension and vibration, abdomen bending, and ejaculation, but not attempted copulation, indicates that activation of neurons in the VNC is sufficient to elicit the former behaviors, whereas attempted copulation requires descending activational input from the brain. These findings are consistent with the results of previous mosaic studies that mapped the foci necessary for particular aspects of courtship behavior [40]–[42]. Thus song production has been mapped to the thorax, and attempted copulation to sites in the anterior suboesophageal ganglion region of the brain and the abdominal ganglion.

### Ordering of courtship steps

Our results also provide insight into the ordering of the steps of courtship. In wild type, the male courtship ritual is largely executed as a dependent action pattern (review [1]). This sequencing of the courtship steps appears to be regulated by *fru^M^*-expressing neurons located in the suboesophageal region of the brain that project through the median bundle: disruption of *fru^M^* expression in just these *fru^M^*-expressing neurons results in the later steps of courtship (wing extension and vibration, licking, and attempted copulation) occurring simultaneously [44]. While there are several models consistent with these observations [44], we favor our previous interpretation that these neurons may normally act as a governor that sets progressively higher thresholds of male excitation to proceed from one step of courtship to the next, thereby ensuring a consistent behavioral sequence.

Interestingly, when we examined the initiation of wing extension and abdomen bending during the shifts from the permissive to the restrictive temperature using either one of the *fru^GAL4^* drivers together with *UAS*-*dTrpA1* in intact males, we found these behaviors were initiated in their normal temporal sequence wherein wing extension always appeared before abdomen bending (and later, attempted copulation). This temporal order of initiation is consistent with the notion that there is a lower activational threshold for wing extension than for abdomen bending. If the median bundle *fru^M^* neurons were responsible for this latency difference (thereby establishing courtship order), it would be expected that the ordered appearance of these behaviors would be lost in headless males (which lack median bundle neurons). Indeed, we found that latencies for wing extension and abdomen bending during the shift from 22°C to 29°C were not significantly different in headless males expressing *fru^M^* driven *dTrpA1*. Consistent with this finding, we also found that activating all *dsx* neurons in *fru^M^* null males induced wing extension and abdomen bending simultaneously, demonstrating that the ordering of courtship steps in our assay is *fru^M^*-dependent as well as brain-dependent.

### Shaping of wing usage

One behavior—bilateral wing extension and vibration—was elicited at a high frequency in response to activation of all *fru^M^*-expressing neurons in intact solitary males, although this behavior is not normally seen in courtship by males of this species. A temporal analysis of the indices for unilateral vs. bilateral wing extension revealed that, following the shift to the restrictive temperature, wing usage was predominantly unilateral for ∼20 min. After that time, wing usage became progressively bilateral and was essentially completely bilateral after ∼30 min. Clyne and Miesenbock [34] also observed an increased frequency of bilateral wing extension after prolonged light activation of *fru^M^*-expressing neurons. These observations suggest that a subset of the *fru^M^* circuitry responsible for wing extension and vibration functions specifically to inhibit bilateral wing extension, and that under conditions of prolonged active wing usage during song performance this inhibition fails.

In contrast, when all *dsx*-expressing neurons were activated in intact solitary males, wing usage was solely unilateral and remained that way throughout the 60 min assay period. Strikingly, headless males with activated *dsx* neurons showed exactly the opposite pattern of wing usage from that seen in intact males of the same genotype: wing usage was almost entirely bilateral at all times. Together, these two findings indicate that in males with *dsx* neurons activated that (1) there are *dsx*-expressing neurons in the VNC capable of driving bilateral wing usage and vibration, and (2) there is active input from the brain that causes wing usage to be unilateral. This finding is consistent with previous fate mapping experiments that identified a posterior dorsal brain focus for unilateral wing extension [40], as well as a recent study showed that lateralized gustatory inputs to male-specific interneurons in the brain ensured unilateral wing extension [47].

Further insight into the control of wing usage comes from the phenotype of *fru^M^* null males in which all *dsx* neurons are activated. In these males, the indices of unilateral and bilateral wing usage are roughly equal throughout the assay period. This result shows that in the absence of wild-type *fru^M^* function there is a “ground state pattern” of wing usage in which unilateral and bilateral usage are equally likely. Further, to account for the differences between the results in Figure 3A–D and Figure 3E, we propose that one of the wild-type functions of *fru^M^* is to inhibit bilateral wing usage, a role that makes sense in that unilateral usage is male-specific, whereas the major bilateral use of wings is in flight.

### Properties of courtship song in males with activated courtship circuitry

Evidence that males with activated *fru^M^* or *dsx* circuitry can sense and respond to external courtship cues came from an examination of recordings of courtship song produced by these males.

Solitary *UAS*-*dTrpA1*/+; *fru^GAL4(D)^*/+ males at the restrictive temperature produced pulse song whose IPIs were significantly longer than those of wild-type male-female pairs. However, when *UAS*-*dTrpA1*/+; *fru^GAL4(D)^*/+ males were paired with female targets, the IPI was reduced toward the level seen in wild-type male-female pairs, indicating that these males could sense and respond to the presence of a female.

Activating the *dsx* circuitry in solitary males via the two *dsx^GAL4^* drivers produced discordant results: *dsx^GAL4(^* ^Δ^ *^2)^* triggered both sine and pulse components as seen in wild type, whereas the *dsx^GAL4(1)^* driver triggered predominantly sine song, with sporadic pulses. However, when we tested *dsx^GAL4(1)^* males with female targets at the restrictive temperature, they showed both sine and pulse song components. Thus, these males were able to sense a female and change the characteristics of their song in response to that. Activating *dsx* neurons at 27°C in solitary *UAS*-*dTrpA1*/+; *dsx^GAL4(^* ^Δ^ *^2)^*/+ males also induced pulse song with elevated IPIs compared to wild-type males, while headless males of this genotype had IPIs that were longer than those of intact males. Similar to what was seen in males with *fru^M^* neurons activated, the presence of a female restored the intact *UAS*-*dTrpA1*/+; *dsx^GAL4(^* ^Δ^ *^2)^*/+ male's IPI to the wild-type level. These results suggest that there are at least two components in the brain that modulate the IPI of courtship song produced by neurons in the ventral nerve cord: (1) certain descending neurons that are responsible for song differences between intact and headless males; and (2) unknown substrates that respond to females and account for differences in the IPIs between solitary males and male-female pairs.

### Function of *fru^M^* and *dsx* neurons in mate recognition and target-directed courtship

Although the courtship behaviors of solitary males with either *fru^M^*- or *dsx-*activated neurons have many similarities, these two types of males behave quite differently when placed with various potential courtship targets. Males with activated *fru^M^* neurons avidly courted intact as well as headless *D. melanogaster* females. These males also robustly courted headless *D. melanogaster* males and headless females of two other Drosophila species, but with CI_target_s that were significantly lower than those with *D. melanogaster* females. This pattern of courtship is quite distinct from that of wild type and other control males, which court *D. melanogaster* females but not headless *D. melanogaster* males or headless females of other species. This difference in response to potential courtship targets indicates that these *fru^M^*-activated males have a lowered threshold for initiating courtship with a potential target (i.e. they robustly court a wider range of targets than does wild type). This could be either because they need less sensory input than wild type to initiate courtship, or because the activation of certain *fru^M^* neurons in these males causes them to perceive certain positive cues that are not in fact there. Yet despite their willingness to direct courtship toward inappropriate targets, *fru^M^*-activated males retain the ability to distinguish appropriate from inappropriate targets when presented with a choice between two such targets. With regard to the dependence of these males on extrinsic sensory cues, we note that their target-directed courtship is not strongly dependent on visual stimuli, since these males also court *D. melanogaster* females as well as inappropriate targets at high levels in the dark. In summary, these results indicate three things: (1) activation of *fru^M^* neurons promotes courtship; (2) males with all *fru^M^* neurons activated still possess the ability to recognize potential courtship targets and direct their efforts towards these targets; and (3) males with all *fru^M^* neurons activated can discriminate conspecific females from other flies as courtship targets.

Assaying the behaviors of *dsx*-activated males toward potential courtship targets produced quite different results as there was no gross affect on the male's ability either to recognize conspecific females and direct courtship to them, or to recognize other potential courtship objects as inappropriate and not court them. These observations suggest that activating *dsx* neurons does not have a significant affect on the male's ability to sense and integrate sensory cues relevant for mate choice.

Our finding that activating all *fru^M^* neurons results in substantial courtship to inappropriate targets suggests that this system might be a part of the courtship circuitry that expresses *fru^M^*, but not *dsx*. To investigate this possibility, we activated *dsx* neurons in males that carried a null mutant combination of *fru^M^* alleles. In this *fru^M^* null background, courtship of inappropriate targets by males with activated *dsx* circuitry appeared elevated. Thus *fru^M^*-expressing neurons are responsible, at least in part, for the ability to distinguish appropriate from inappropriate courtship objects.

It is also worth noting that recognition of potential courtship targets by *UAS-dTrpA1/+*; *dsx^GAL4(^* ^Δ^ *^2)^/+* males that are null for *fru^M^* appears to be largely vision (motion) dependent, as these males do not court headless *D. melanogaster* females (which move little), nor do they court intact *D. melanogaster* females in the dark.

### Courtship in the absence of *fru^M^* function

It has been proposed that the FRU^M^ proteins function to specify the neuronal circuitry subserving male courtship behavior [2], [7], and there is a significant body of data interpreted as either supporting or being consistent with this view (reviews [2]–[6], [8], [9]). Most notable in this regard have been the demonstrations that the expression of *fru^M^* in the appropriate cells is both necessary and sufficient for male courtship behavior [4], [12], [20]. We were therefore more than a little surprised to discover that intact males that were null for *fru^M^* (*fru^LexA^/fru^4–40^*) and had *dsx* neurons activated produced courtship outputs as avidly as males that were wild type for *fru^M^* and had *dsx* neurons activated (CI_output_s ∼90%; Figure 6E). Moreover, these *fru^M^* null males with *dsx* neurons activated directed courtship toward intact conspecific females robustly (CI_target_>50%), although not as avidly as males that were wild type for *fru^M^* and had *dsx* neurons activated (CI_target_>80%). These results reveal the presence of a neural circuit in the CNS containing *dsx*-expressing CNS neurons (and potentially other neurons downstream of the *dsx*-expressing neurons) that is capable of eliciting most of the steps comprising courtship in response to activation of *dsx* neurons independent of *fru^M^* function.

Additional evidence pointing toward the existence of a *fru^M^*-independent pathway by which courtship can be elicited are the findings that males carrying null combinations of *fru* alleles show low levels of male-male courtship when multiple males are grouped together (CIs ∼5–20%) [48]–[50]. Males of these genotypes do not show courtship behavior when paired together with females [48]. As multiple null *fru* genotypes, including those with molecularly defined rearrangements, exhibit male-male courtship, it is unlikely that these are misclassified hypomorphic mutants that express *fru^M^* at a reduced level. Thus, these observations provide additional evidence that at least some aspects of male courtship can be elicited in the absence of *fru^M^*.

From consideration of the above findings, several implications arise with regard to the nature of a potential *fru^M^*-independent pathway through which courtship can be elicited. First, while these findings indicate that the functioning of such a pathway does not depend on *fru^M^* function, they do not preclude the possibility that some or all of the neurons in this pathway express *fru^M^* in wild type; rather, if *fru^M^* is expressed in these neurons, its function is not needed in these neurons for the elicitation of the observed behaviors in our experiments. By extension, the functional properties of this circuit that allow it to be used to elicit male courtship in response to the activation of *dsx* neurons must depend on some gene(s) other than *fru^M^*. Whether *dsx* might provide this function is an attractive idea that will have to await future examination.

The notion that there may be more than one neuronal pathway for the elicitation of male courtship behaviors has attractive features from an evolutionary perspective. First, parallel or only partially overlapping neuronal pathways that are each formed by the actions of different genes would confer redundancy to the courtship circuitry and thereby afford robustness in the face of random mutations and developmental events that would otherwise pose a great threat to a simple linear pathway of neurons. Given that the sine-qua-non of male reproductive fitness depends on the proper execution of courtship, we envision that pathway redundancy would be favorable.

Indeed, in recent experiments screening random *GAL4* enhancer trap lines driving *UAS-fru^MIR^*, an RNAi construct directed against *fru^M^* transcripts, for a variety of courtship behavior phenotypes, we found that male behavioral sterility was quite rare compared to other phenotypes such as male-male courtship/chaining, and changes in the latency of wing extension (Meissner et al., in preparation). From this finding, we suggest that at least certain aspects of the courtship circuitry might be redundant.

Second, the primitive ancestors of the Diptera are thought to be non-social flies, and it has been proposed that their “courtship” was similar to that of contemporary solitary flies like Musca and Calliphora (reviews [51], [52]). Courtship behavior in these species is very dependent on vision, as potential mates are generally only encountered during flight. Indeed, the males in these species often have specializations in their visual system that serve to enhance motion tracking [51], [53]–[56]. Courtship behaviors by these males consist of identifying a moving object of about the right size and speed, then following and grabbing it during flight. At this juncture, conspecific females are distinguished from other objects and attempted mating ensues or the inappropriate object is released. In this regard, it is perhaps of more than passing interest to note that the courtship behavior towards females displayed by *fru^M^* null males in which *dsx*-expressing neurons have been activated is almost entirely based on vision (motion cues), and thus is strikingly different from wild type in its sensory modality dependence. Perhaps the neuronal pathway being utilized to direct courtship in these males is descended from the visual-based courtship behavior system of the ancestral Diptera.

Thus, in the lineage leading to *D. melanogaster*, a neural circuitry capable of integrating information from multiple sensory modalities would have evolved under the direction of *fru^M^*, which is fairly broadly conserved in insects [57], to orchestrate the relatively elaborate multi-step behaviors that comprise modern day male courtship.

## Materials and Methods

### Fly stocks

Flies used in this study include wild-type *Canton S* (*wtcs*), *UAS*-*dTrpA1* [35], *fru^GAL4(B)^* [12], *fru^GAL4(D)^* [13], *fru^LexA^* [32], *fru^4–40^*, *dsx^GAL4(1)^* [19] and *dsx^GAL4(^* ^Δ^ *^2)^* (see below). All crosses to generate flies for behavioral testing were performed at 18°C or 22°C.

### Molecular biology

To generate the *dsx^GAL4(^* ^Δ^ *^2)^* targeted insertion of *GAL4* into the *dsx* gene, the *dsx* 2.8-kb 5′ homology arm and *GAL4* coding sequence were the same as in [19], but a new 3′ homology arm was designed to allow deletion of exon 2 coding sequence in the course of homologous recombination. The new 2.7-kb 3′ homology arm, extending between genomic sequences GCAATATTGGCACTCAGCTATTATC and CACGTTCGATATTGAGTTGGGTGAA in the *dsx* second intron, was PCR-amplified from genomic DNA prepared with the DNeasy Tissue Kit (Qiagen). The transcriptional stop cassette containing the *SV40 poly-A* sequence in tandem with the *D. melanogaster* α*-tubulin 84B 3′UTR* was from [13]. All DNA fragments were generated by PCR using AccuPrime Supermix (Invitrogen) and were sequenced. Using restriction endonuclease sites added to the 5′ ends of the PCR primers, these fragments were cloned in the linear order of *dsx* 5′ arm-*GAL4-SV40 poly-A/*α*-tubulin 84B 3′ UTR*-*dsx* 3′ arm into p*P{WhiteOut2}* (gift of Jeff Sekelsky) to make p*P{WO2-dsx*-*GAL4*-*stop*-Δ*2}*. Details available upon request.

### Transgensis and homologous recombination

p*P{WO2-dsx*-*GAL4*-*stop*-Δ*2}* transgenics were made by *P* element-mediated germline transformation using standard methods (Rainbow Transgenic Flies, Inc.), and six independent integrant lines were isolated to serve as donors of the *dsx*-*GAL4*-*stop*-Δ*2* DNA substrate for homologous recombination [58]. Donors were crossed to a line containing heat-shock-inducible FLP recombinase and I-SceI endonuclease transgenes [58] and larvae were heat shocked for 1 hour at 37°C on days 3 and 4 of development. ∼1500 female F1 progeny containing all three elements were crossed to *UAS-mCD8::GFP* males and the F2 progeny screened for candidates with changes in the GFP expression pattern relative to the donors alone. Two candidate lines producing intersexual progeny when crossed to *dsx^1^* were PCR-tested using the 5′ genomic and *GAL4* primers, GTGTGTGAGGCTGCCTATGTACTAG and ATGCTTGTTCGATAGAAGACAGTAG, respectively, and the 3′ genomic and *a-tubulin 84B 3′ UTR* primers, GAAAGTCGCAGTTTCCTACTGATAC and TGTGTCAGTCCTGCTTACAGGAACG, respectively. Using these primer pairs, insert-specific PCR products were generated for the 5′ and 3′ ends of the inserted *GAL4-stop* sequences for one of the candidates. This *dsx^GAL4(^* ^Δ^ *^2)^* chromosome was balanced over the *TM6B* balancer.

### Behavior

Male progenies were collected within 12 hours after eclosion and housed individually for 4–7 days at 18°C or 22°C. Flies used as courtship targets were collected in the same way but group housed at 22°C. All flies were briefly cooled on ice and loaded to behavioral chambers (diameter: 1 cm; height: 2 mm) at room temperature (22°C) at least 1 hour before transferring to incubators (either 22°C, 25°C, 27°C or 29°C) where videos were taken. For solitary males (Figure 1–3), videos were taken immediately after the transfer; for paired courtship at 27°C (Figure 5–6), flies were firstly warmed up for ∼10 min and then introduced to each other.

In our initial experiments, 29°C was used as the restrictive temperature. However, we found that solitary *UAS-dTrpA1/+*; *fru^GAL4^/+* males began to fall over and were on their backs after ∼15 min at this temperature, where they appeared to be still almost continuously carrying out courtship behaviors (wing extension, abdomen bending, see Movie S1), and were dead by ∼4 hours. Solitary *UAS-dTrpA1/+*; *dsx^GAL4^/+* males were also dead in ∼5 hours. We surmise that their monomaniacal pursuit of sex overrode multimodal integration centers that would normally prevent them from killing themselves in pursuit of sex. To enable longer observation periods we switched to 27°C for many experiments.

To test headless males, flies were decapitated at least 1 hour before testing. To control the potential effect of severed descending axons from the head, headless males (*UAS-dTrpA1/+*; *fru^GAL4(D)^/+* and *UAS-dTrpA1/+*; *dsx^GAL4(^* ^Δ^ *^2)^/+*) were kept in humidity at 18°C and tested at 1d, 2d, 3d or 4d after a shift to 29°C; 100% of solitary males tested still displayed high levels of wing extension and abdomen bending (for two genotypes and each time point, at least 12 males that were standing well were tested; ejaculation was not scored at these time points).

In order to initiate courtship behavior in solitary males quickly, we also used another temperature control system (AHP-301CPV, TECA Corp.) The top of the system is a metal plate whose temperature is precisely controlled. We put the behavioral chamber with flies on the plate (set as 29°C) and quickly remove the barrier, allowing flies to directly contact the 29°C plate.

To score courtship behaviors in solitary males (Figure 1 and Table S1, S2), we firstly took video for 15 min, and then immediately checked the behaving flies under a microscope for ∼1 min, especially for proboscis extension and ejaculation. Wing extension index (Figure 2–3 and Figure S4, S5) in solitary males is defined as percentage of observation time during which males display wing extension. Abdomen bending index is the percentage of observation time during which males bend their abdomen (Figure 2 and Figure S4). The distinction between abdomen bending and attempted copulation is the latter is a momentary thrusting action during which a male fully curls its abdomen (see Movie S2).

To score courtship behavior in males with targets (Figure 5–6), two courtship indices were used: (1) Index of total courtship outputs (CI_output_), which is the percentage of time during which males display any of the courtship rituals, regardless of whether they are directed at a courtship target or not; (2) Index of courtship to targets (CI_target_, a fraction of CI_output_), which is the percentage of time during which males direct any of the courtship rituals to targets. CI_target_ and CI_output_ will only be used in males when *fru^M^* or *dsx* neurons are activated via *dTrpA1* at 27°C. Since control males do not court without targets, the CI_target_ and CI_output_ are the same and will simply refer to CI. All indices were analyzed manually using LifeSongX [59].

### Courtship song recording and analyzing

Courtship songs were recorded for 10–15 min using an optical microphone that detects wing movements associated with sine and pulse songs (Lott, Simpson, et al., unpublished).

Audio files were loaded into LifeSongX and analyzed manually. Only song samples in the first 3 min were used, except for Figure S3G, S3I and S3J that are samples from later times. To analyze IPI of the pulse song, only pulse trains with at least 6 pulses were used; for sine song frequency, only samples with at least 20 cycles were used. For each fly, the mean value of IPIs or sine song frequencies was used for statistics, thus the number of statistical samples is the number of flies.

## Supporting Information

Figure S1 **Labeling of sperm in ejaculated substance.** Solitary males with all *fru^M^* (*UAS*-*dTrpA1*/*don juan-GFP*; *fru^GAL4^*/+) or all *dsx* (*UAS*-*dTrpA1*/*don juan-GFP*; *dsx^GAL4^*/+) neurons activated at 29°C were checked under fluorescent microscope after ejaculation. The green signals indicate sperm.
(TIF)

Figure S2 **Activation of** ***fru^M^-*** **expressing neurons induces courtship song in females.** (A) Activation of neurons that are *fru^M^* counterparts (*UAS-dTrpA1/+*; *fru^GAL4^/+*) initiated unilateral wing extension in solitary females at 29°C. (B–C) Song samples of solitary *UAS-dTrpA1/+*; *fru^GAL4^/+*females at 29°C. For both *fru^GAL4(B)^* (B) and *fru^GAL4(D)^* (C), only pulse song was detected. Scale bars as indicated.
(TIF)

Figure S3 **Courtship song samples of males with activation of all** ***fru^M^*** **or** ***dsx*** **neurons.** (A) A wild-type (*wtcs*) male paired with a *wtcs* female at 27°C produced alternating sine and pulse songs. (B–D) Activating all *dsx* neurons in solitary males using *dsx^GAL4(1)^* produced dominant sine song (B); however, the introduction of female targets restored the pulse component at both 22°C (C) and 27°C (D). (E) Activating all *dsx* neurons in solitary males using *dsx^GAL4(^* ^Δ^ *^2)^* produces both sine and pulse components. (F–J) Courtship samples from solitary males with all *fru^M^* neurons activated. These males first showed separate sine and pulse songs (F and H), but after continuous activation for several minutes, the two components began to occur simultaneously (gray boxes in G and I). The gray box in I is zoomed in as indicated in J. Scale bars as indicated.
(TIF)

Figure S4 **Kinetics of wing extension and abdomen bending in headless males.** Wing extension and abdomen bending in headless males were analyzed independently in every minute after transfer from 22°C to 29°C for 15 min. (A–D) Indices of wing extension (blue) and abdomen bending (red) are shown over time for activation driven by *fru^GAL4(D)^* (A), *fru^GAL4(B)^* (B), *dsx^GAL4(1)^* (C) and *dsx^GAL4(^* ^Δ^ *^2)^* (D). *n* = 8–10 for each. Error bars indicate SEM.
(TIF)

Figure S5 **Wing extension patterns in intact and headless males (supplementary to** **Figure 3** **).** For each male, indices for either unilateral wing extension (white) or bilateral wing extension (gray) were calculated in every 5 min for 1 hour at 27°C. (A–B) Wing extension indices in intact (A) and headless (B) *UAS-dTrpA1/+*; *fru^GAL4(B)^/+* males at 27°C. (C–D) Wing extension indices in intact (C) and headless (D) *UAS-dTrpA1/+*; *dsx^GAL4(1)^/+* males at 27°C. *n* = 8–10 for each. Error bars indicate SEM.
(TIF)

Movie S1 **Courtship behavior displayed by a solitary** ***UAS-dTrpA1/+*** **;** ***fru^GAL4(B)^/+*** **male at 29°C.** The male first showed wing extension (stage 1), and later other steps such as abdomen bending (stage 2), then frequent attempted copulation, licking and even ejaculation (stage 3), finally the male fell over and was on its back (stage 4).
(MOV)

Movie S2 **Abdomen bending and attempted copulation shown by a solitary** ***UAS-dTrpA1/+*** **;** ***fru^GAL4(B)^/+*** **male at 29°C.**
(MOV)

Movie S3 **Courtship behavior displayed by a solitary** ***UAS-dTrpA1/+*** **;** ***dsx^GAL4(^*** ^Δ^ *^2)^/+* **male at 29°C.** The male first showed wing extension (stage 1), and later abdomen bending (stage 2), then frequent attempted copulation and licking (stage 3).
(MOV)

Movie S4 **Courtship behavior displayed by a solitary** ***UAS-dTrpA1/+*** **;** ***dsx^GAL4(^*** ^Δ^ *^2)^* **,** ***fru^LexA^/fru^4–40^*** **male at 29°C.** Wing extension and abdomen bending were elicited almost simultaneously.
(MOV)

Movie S5 **A** ***UAS-dTrpA1/+*** **;** ***fru^GAL4(B)^/+*** **male copulates with a** ***wtcs*** **female at 27°C.** The female was introduced to the male after 5–10 min at 27°C when the male already showed wing extension and abdomen bending.
(MOV)

Movie S6 ***UAS-dTrpA1/+*** **;** ***dsx^GAL4(^*** ^Δ^ *^2)^/+* **male courtship in** ***fru^M+^*** **and** ***fru^M−^*** **background** ***s*** **.** The first half of the movie shows a *UAS-dTrpA1/+*; *dsx^GAL4(^* ^Δ^ *^2)^/+* male courting a headless female persistently at 27°C; while the second half shows a *UAS-dTrpA1/+*; *dsx^GAL4(^* ^Δ^ *^2)^*, *fru^LexA^/fru^4–40^* male displaying courtship behavior, but not to the headless female at 27°C. Headless females were introduced to the males after ∼10 min at 27°C when the male already displayed wing extension and abdomen bending.
(MOV)

Table S1 **Behavioral outputs of solitary males at 22°C, 25°C and 27°C for 15 min.** Behaviors were scored for 15 min in solitary males with indicated genotypes after transfer from 22°C to 25°C or 27°C. No courtship-like behavior was observed at 22°C in all genotypes. Wing extension was induced at 25°C by activating all *fru^M^* but not *dsx* neurons; while abdomen bending and attempted copulation were not observed at 25°C. At 27°C, wing extension and abdomen bending could be faithfully initiated in solitary males using either *fru^GAL4^* or *dsx^GAL4^*. Attempted copulation was also found in solitary males by activating all *fru^M^* but not *dsx* neurons at 27°C.
(DOCX)

Table S2 **Behavioral outputs of solitary males at 29°C for 15 min.** Behaviors were scored for 15 min in solitary males with indicated genotypes after transfer from 22°C to 29°C. No courtship-like behavior was observed at 29°C in solitary males with *UAS-dTrpA1* or *GAL4* (for either *fru^GAL4(D)^*, *fru^GAL4(B)^*, *dsx^GAL4(1)^* or *dsx^GAL4(^* ^Δ^ *^2)^*) alone. All solitary *UAS-dTrpA1/+*; *dsx^GAL4(^* ^Δ^ *^2)^*, *fru^LexA^/fru^4–40^* males displayed wing extension (either unilateral or bilateral), proboscis extension and abdomen bending within 15 min at 29°C; however, none of these males showed attempted copulation in 15 min. 2 out of 24 males of this genotype ejaculated in 30 min at 29°C.
(DOCX)

Table S3 **Comparison of expression patterns for** ***dsx^GAL4(1)^*** **and** ***dsx^GAL4(^*** ^Δ^ *^2)^* **.** Comparison of expression patterns generated by each of the two *dsx^GAL4^* lines driving the *UAS-mCD8::GFP* membrane-bound GFP reporter. A comparison is shown only for tissues that were specifically examined in each *dsx^GAL4^* line. “+” indicates expression was observed; “−” indicates expression was not detected; “− (few cells)” indicates very few cells in a tissue were seen to express the reporter.
(DOCX)
