# Supplementary figures and images for: Turning Males On: Activation of Male Courtship Behavior in Drosophila melanogaster
Source: PLoS One. 2011 Jun 22;6(6):e21144. doi: 10.1371/journal.pone.0021144 (PMC3120818; doi:10.1371/journal.pone.0021144)

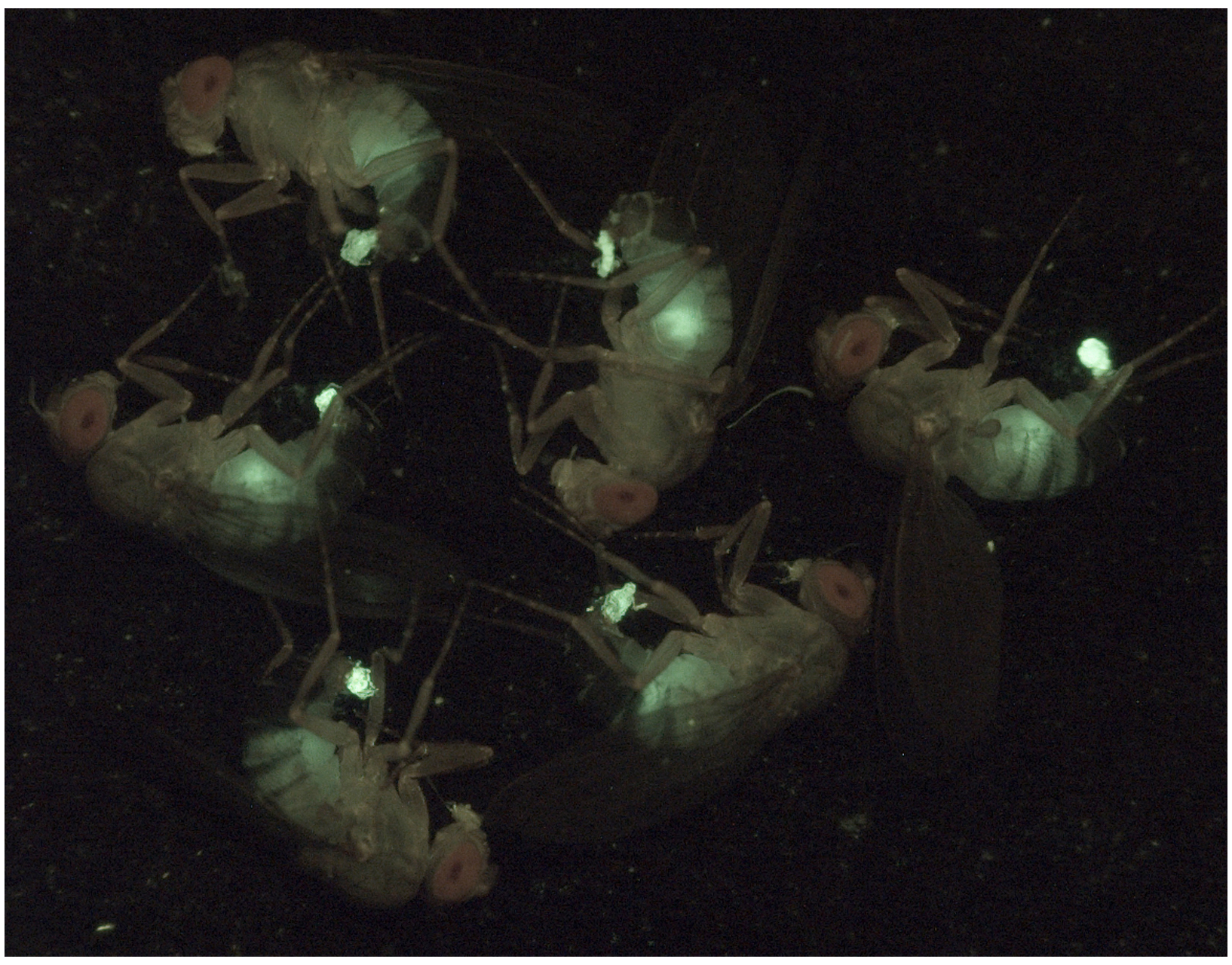

Supplement: Figure S1 — Labeling of sperm in ejaculated substance. Solitary males with all fruM (UAS-dTrpA1/don juan-GFP; fruGAL4/+) or all dsx (UAS-dTrpA1/don juan-GFP; dsxGAL4/+) neurons activated at 29°C were checked under fluorescent microscope after ejaculation. The green signals indicate sperm. (TIF) [file pone.0021144.s001.tif]

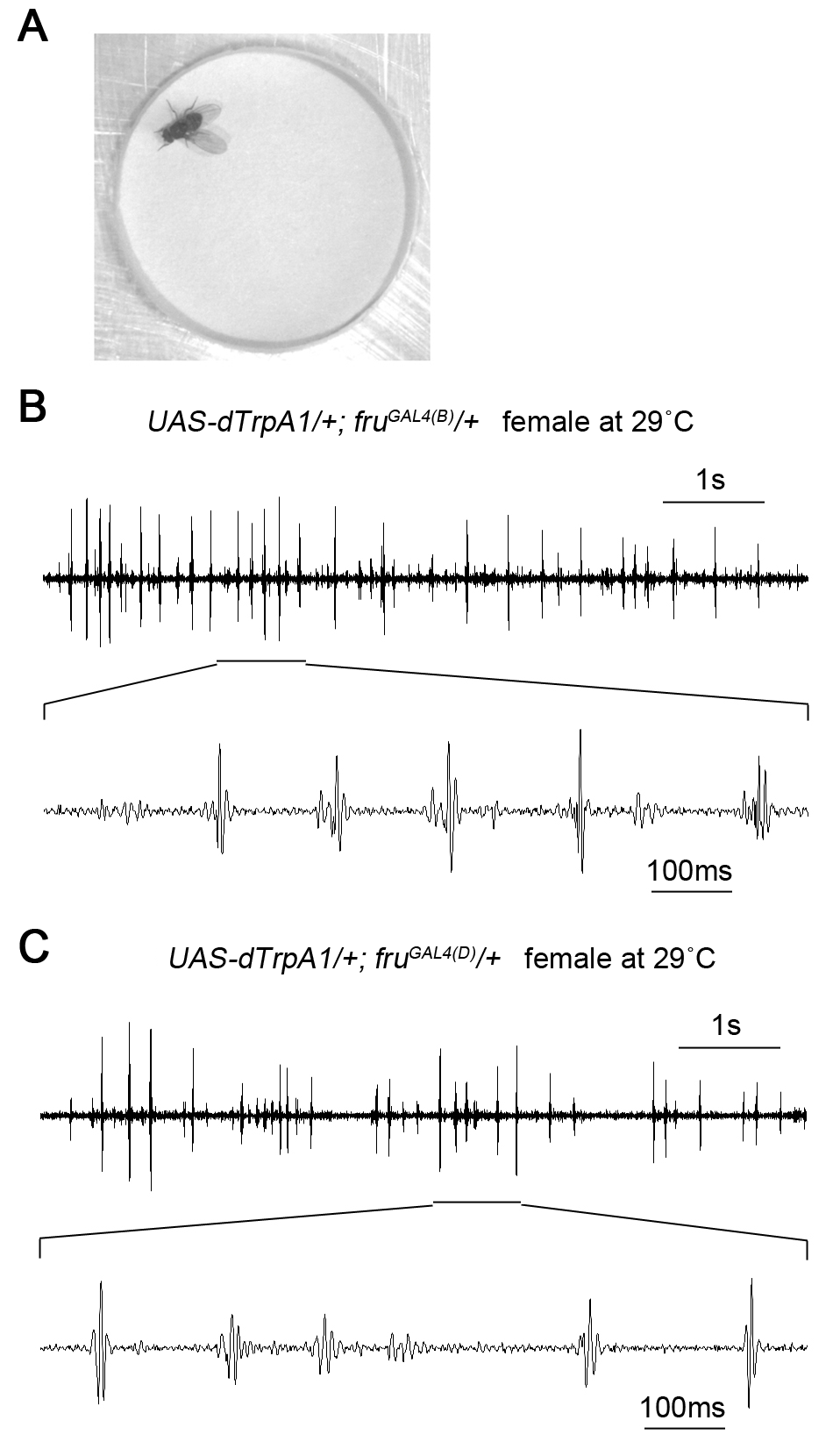

Supplement: Figure S2 — Activation of fruM- expressing neurons induces courtship song in females. (A) Activation of neurons that are fruM counterparts (UAS-dTrpA1/+; fruGAL4/+) initiated unilateral wing extension in solitary females at 29°C. (B–C) Song samples of solitary UAS-dTrpA1/+; fruGAL4/+females at 29°C. For both fruGAL4(B) (B) and fruGAL4(D) (C), only pulse song was detected. Scale bars as indicated. (TIF) [file pone.0021144.s002.tif]

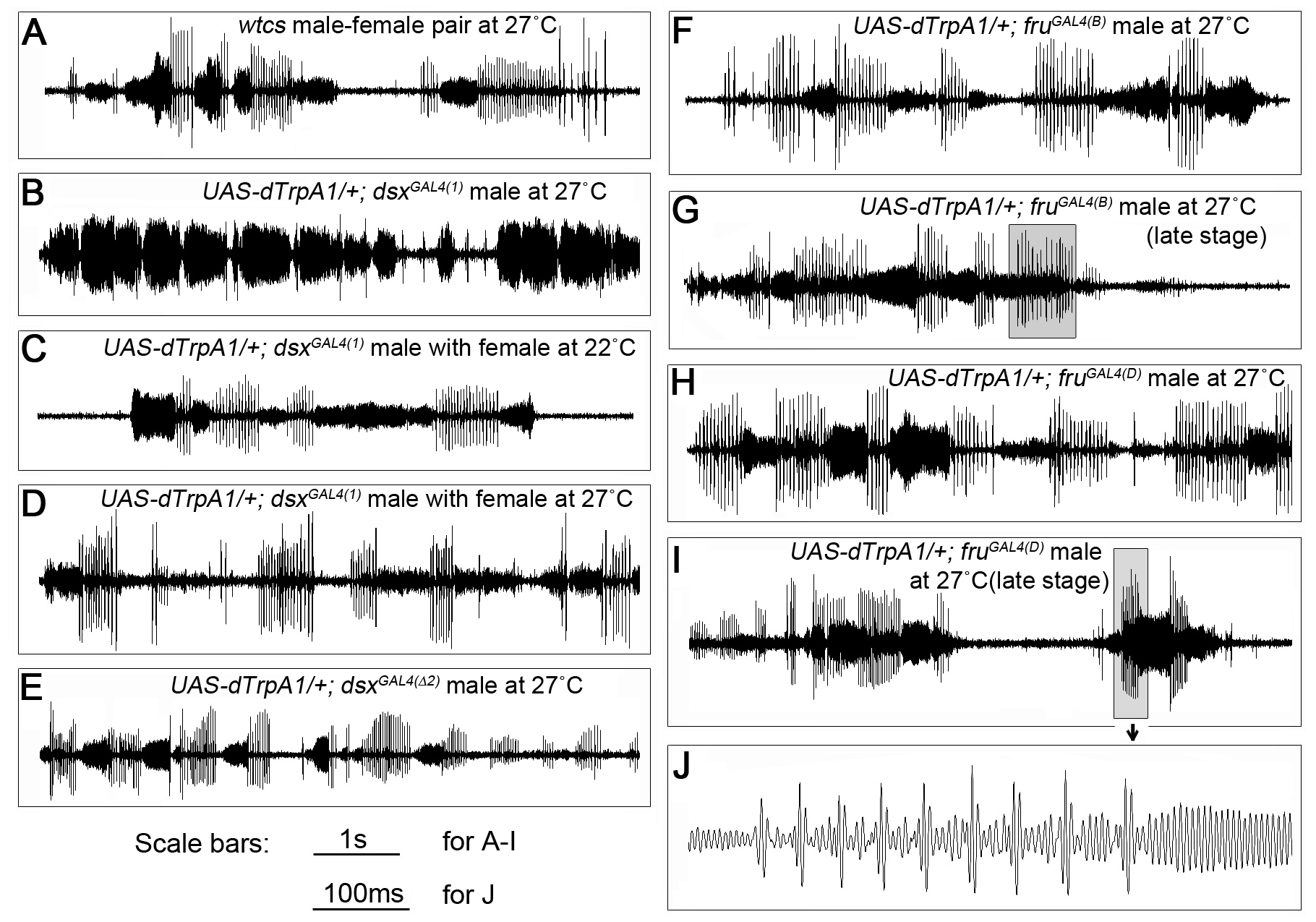

Supplement: Figure S3 — Courtship song samples of males with activation of all fruM or dsx neurons. (A) A wild-type (wtcs) male paired with a wtcs female at 27°C produced alternating sine and pulse songs. (B–D) Activating all dsx neurons in solitary males using dsxGAL4(1) produced dominant sine song (B); however, the introduction of female targets restored the pulse component at both 22°C (C) and 27°C (D). (E) Activating all dsx neurons in solitary males using dsxGAL4( Δ 2) produces both sine and pulse components. (F–J) Courtship samples from solitary males with all fruM neurons activated. These males first showed separate sine and pulse songs (F and H), but after continuous activation for several minutes, the two components began to occur simultaneously (gray boxes in G and I). The gray box in I is zoomed in as indicated in J. Scale bars as indicated. (TIF) [file pone.0021144.s003.tif]

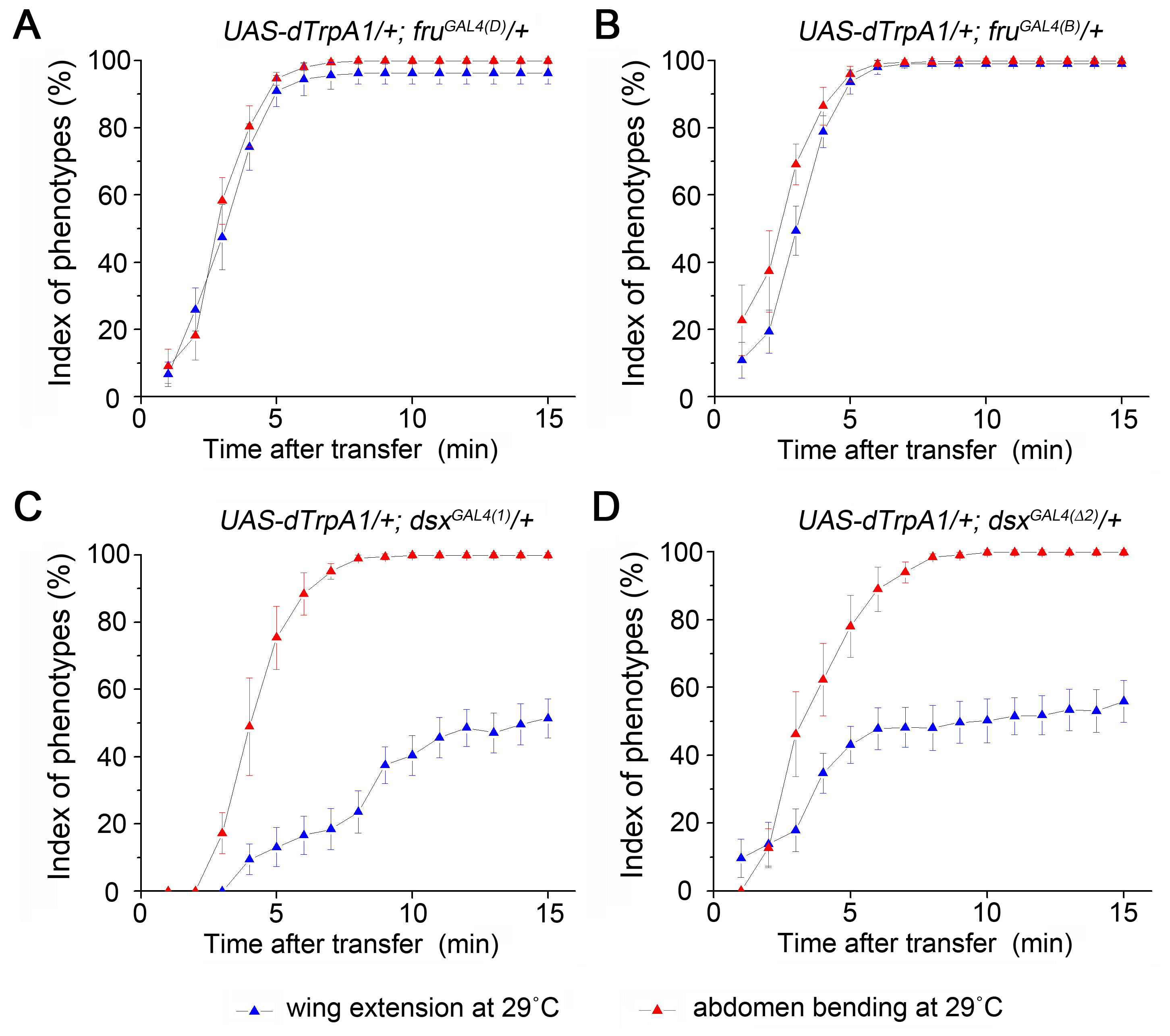

Supplement: Figure S4 — Kinetics of wing extension and abdomen bending in headless males. Wing extension and abdomen bending in headless males were analyzed independently in every minute after transfer from 22°C to 29°C for 15 min. (A–D) Indices of wing extension (blue) and abdomen bending (red) are shown over time for activation driven by fruGAL4(D) (A), fruGAL4(B) (B), dsxGAL4(1) (C) and dsxGAL4( Δ 2) (D). n = 8–10 for each. Error bars indicate SEM. (TIF) [file pone.0021144.s004.tif]

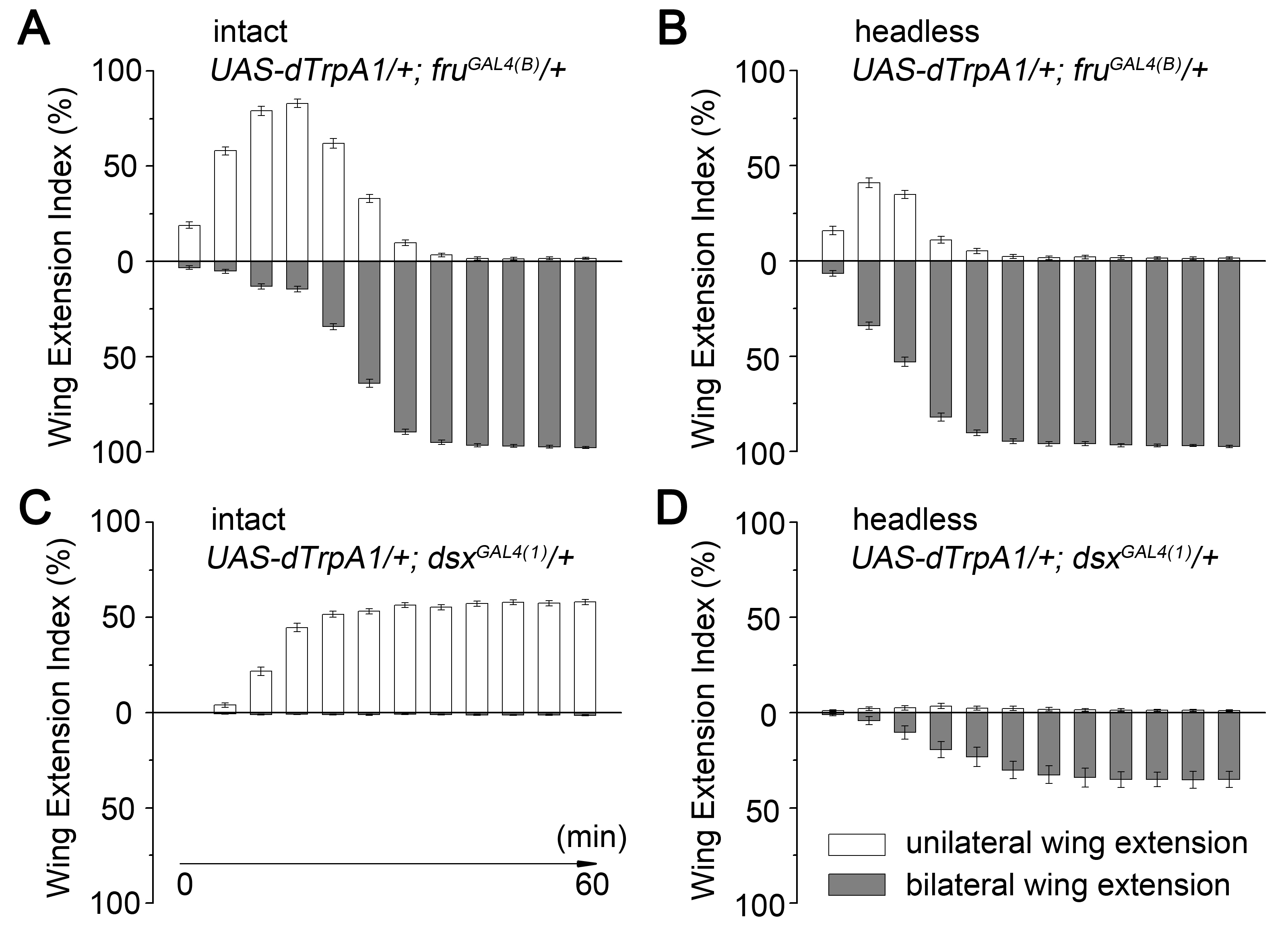

Supplement: Figure S5 — Wing extension patterns in intact and headless males (supplementary to Figure 3 ). For each male, indices for either unilateral wing extension (white) or bilateral wing extension (gray) were calculated in every 5 min for 1 hour at 27°C. (A–B) Wing extension indices in intact (A) and headless (B) UAS-dTrpA1/+; fruGAL4(B)/+ males at 27°C. (C–D) Wing extension indices in intact (C) and headless (D) UAS-dTrpA1/+; dsxGAL4(1)/+ males at 27°C. n = 8–10 for each. Error bars indicate SEM. (TIF) [file pone.0021144.s005.tif]
