# Supplementary material for: Turning Males On: Activation of Male Courtship Behavior in Drosophila melanogaster
Source: PLoS One. 2011 Jun 22;6(6):e21144. doi: 10.1371/journal.pone.0021144 (PMC3120818; doi:10.1371/journal.pone.0021144)
Supplement: Table S1 — Behavioral outputs of solitary males at 22°C, 25°C and 27°C for 15 min. Behaviors were scored for 15 min in solitary males with indicated genotypes after transfer from 22°C to 25°C or 27°C. No courtship-like behavior was observed at 22°C in all genotypes. Wing extension was induced at 25°C by activating all fruM but not dsx neurons; while abdomen bending and attempted copulation were not observed at 25°C. At 27°C, wing extension and abdomen bending could be faithfully initiated in solitary males using either fruGAL4 or dsxGAL4. Attempted copulation was also found in solitary males by activating all fruM but not dsx neurons at 27°C. (DOCX) [file pone.0021144.s012.docx]

**Table S1. Behavioral outputs of solitary males at 22˚C, 25˚C and 27˚C for 15 min.**

|  | *UAS-dTrpA1/+; fru^GAL4(D)^/+* | | | *UAS-dTrpA1/+; fru^GAL4(B)^/+* | | | *UAS-dTrpA1/+; dsx^GAL4(1)^/+* | | | *UAS-dTrpA1/+; dsx^GAL4(∆2)^/+* | | |
| --- | --- | --- | --- | --- | --- | --- | --- | --- | --- | --- | --- | --- |
| Phenotypes | 22˚C | 25˚C | 27˚C | 22˚C | 25˚C | 27˚C | 22˚C | 25˚C | 27˚C | 22˚C | 25˚C | 27˚C |
| Wing extension | 0/24 | 11/24 | 24/24 | 0/24 | 7/24 | 24/24 | 0/24 | 0/24 | 19/24 | 0/24 | 0/24 | 24/24 |
| Abdomen bending | 0/24 | 0/24 | 24/24 | 0/24 | 0/24 | 24/24 | 0/24 | 0/24 | 10/24 | 0/24 | 0/24 | 19/24 |
| Attempted copulation | 0/24 | 0/24 | 20/24 | 0/24 | 0/24 | 22/24 | 0/24 | 0/24 | 0/24 | 0/24 | 0/24 | 0/24 |
