# Supplementary material for: Turning Males On: Activation of Male Courtship Behavior in Drosophila melanogaster
Source: PLoS One. 2011 Jun 22;6(6):e21144. doi: 10.1371/journal.pone.0021144 (PMC3120818; doi:10.1371/journal.pone.0021144)
Supplement: Table S2 — Behavioral outputs of solitary males at 29°C for 15 min. Behaviors were scored for 15 min in solitary males with indicated genotypes after transfer from 22°C to 29°C. No courtship-like behavior was observed at 29°C in solitary males with UAS-dTrpA1 or GAL4 (for either fruGAL4(D), fruGAL4(B), dsxGAL4(1) or dsxGAL4( Δ 2)) alone. All solitary UAS-dTrpA1/+; dsxGAL4( Δ 2), fruLexA/fru4–40 males displayed wing extension (either unilateral or bilateral), proboscis extension and abdomen bending within 15 min at 29°C; however, none of these males showed attempted copulation in 15 min. 2 out of 24 males of this genotype ejaculated in 30 min at 29°C. (DOCX) [file pone.0021144.s013.docx]

**Table S2. Behavioral outputs of solitary males at 29˚C for 15 min.**

|  | *UAS-dTrpA1/+* | *fru^GAL4(D)^/+* | *fru^GAL4(B)^/+* | *dsx^GAL4(1)^/+* | *dsx^GAL4(∆2)^/+* | *UAS-dTrpA1/+; dsx^GAL4(∆2)^,fru^LexA^/fru^4-40^* |
| --- | --- | --- | --- | --- | --- | --- |
| Phenotypes | | | | | | |
| Unilateral  wing extension | 0/24 | 0/24 | 0/24 | 0/24 | 0/24 | 24/24 |
| Bilateral  wing extension | 0/24 | 0/24 | 0/24 | 0/24 | 0/24 | 24/24 |
| Proboscis  Extension | 0/24 | 0/24 | 0/24 | 0/24 | 0/24 | 24/24 |
| Abdomen  bending | 0/24 | 0/24 | 0/24 | 0/24 | 0/24 | 24/24 |
| Attempted  copulation | 0/24 | 0/24 | 0/24 | 0/24 | 0/24 | 0/24 |
| Ejaculation | 0/24 | 0/24 | 0/24 | 0/24 | 0/24 | 2/24 in 30 min |
