# Supplementary material for: Turning Males On: Activation of Male Courtship Behavior in Drosophila melanogaster
Source: PLoS One. 2011 Jun 22;6(6):e21144. doi: 10.1371/journal.pone.0021144 (PMC3120818; doi:10.1371/journal.pone.0021144)
Supplement: Table S3 — Comparison of expression patterns for dsxGAL4(1) and dsxGAL4( Δ 2) . Comparison of expression patterns generated by each of the two dsxGAL4 lines driving the UAS-mCD8::GFP membrane-bound GFP reporter. A comparison is shown only for tissues that were specifically examined in each dsxGAL4 line. “+” indicates expression was observed; “−” indicates expression was not detected; “− (few cells)” indicates very few cells in a tissue were seen to express the reporter. (DOCX) [file pone.0021144.s014.docx]

**Table S3. Comparison of expression patterns for *dsx^GAL4(1)^* and *dsx^GAL4(∆2)^.***

| **Region** | ***dsx^GAL4(1)^*** | ***dsx^GAL4(∆2)^*** |
| --- | --- | --- |
|  |  |  |
| **late 3rd instar tissues:** |  |  |
|  |  |  |
| genital imaginal disc | + | + |
| foreleg imaginal disc | + | + |
| eye-antennal imaginal disc | + | + |
|  |  |  |
| somatic cells of gonad | + | + |
|  |  |  |
| fat body | + | + |
|  |  |  |
| **adult tissues:** |  |  |
|  |  |  |
| foreleg gustatory sensilla | + | + |
| foreleg chordotonal organ | - | + |
| foreleg muscle | + | + |
| tibial mechanosensory sensilla | - | + |
|  |  |  |
| second leg sensilla | - (few cells) | + |
| third leg sensilla | - (few cells) | + |
|  |  |  |
| anterior wing margin | - | + |
|  |  |  |
| Brain | + | + |
| VNC | + | + |
